# Study of the Compositional, Microbiological, Biochemical, and Volatile Profile of Red-Veined Cheese, an Internal *Monascus*-Ripened Variety

**DOI:** 10.3389/fnut.2021.649611

**Published:** 2021-04-16

**Authors:** Jingkai Jiao, Zhiqiang Zheng, Zhenmin Liu, Chunping You

**Affiliations:** State Key Laboratory of Dairy Biotechnology, Shanghai Engineering Research Center of Dairy Biotechnology, Dairy Research Institute, Bright Dairy & Food Co., Ltd., Shanghai, China

**Keywords:** internal mold-ripened cheese, red koji powder, *Monascus*, sandwich inoculation, ripening, proteolysis, lipolysis, volatile compounds

## Abstract

Red-Veined Cheese is an experimental internal mold-ripened cheese using red koji powder as the red starter. The objective of this study was to characterize the physicochemical parameters, microorganisms, proteolysis, lipolysis, and volatile profile of the cheese during 33 days of ripening. The gross composition was 56.7% (w/w) for total solids, 45.8% (w/w) for fat-in-dry matter, 2.58% (w/w) for salt content and 37.8% (w/w) for protein. The pH increased from 4.88 to 5.23 during ripening. The *Monascus* density first rose and then fell, while total mesophilic bacteria count declined steadily throughout the maturation. Proteolysis degree in experimental group was significantly higher than in control group without inoculation of red koji powder. Analysis of the fatty acid profile showed that the internal *Monascus*-fermentation also promoted lipolysis. A total of 63 volatile compounds, including 12 ketones, 14 alcohols, 15 acids, 13 esters, 5 aldehydes, 3 lactones and 1 phenol, were identified by gas chromatography-mass spectrometry (GC–MS) coupled with headspace solid-phase microextraction (HS-SPME). The main chemical groups of volatile compounds were ketones, alcohols, acids and esters whereas aldehydes, lactones, and phenols represented only minor components. At the end of ripening, the levels of ketones, alcohols, esters were significantly higher in the experimental cheese compared with the control. Our results therefore show that the internal *Monascus*-fermentation is not only a suitable technology to form red veins in the paste but also an effective method to impact the composition of volatile compounds in cheese, which can thus distinguish it from other internal mold-ripened cheeses and surface-ripened *Monascus*-fermented cheese.

## Introduction

Cheese is one of the most diverse varieties of dairy products. It has become an important contributor to nutritional intake in public health interests because it contains abundant nutrients for the human body including proteins, fat, bioactive peptides, amino acids, free fatty acids and etc (1, 2). With growing public awareness of health, consumer demand for functional dairy products put forward higher requirements. There is an urgent need to develop healthier, more diversified new cheese products to meet the market demand.

Red koji-mold (*Monascus* sp.) is a well-known industrially mold that produces valuable metabolites such as lovastatin, γ-aminobutyric acid, polysaccharides and etc. (3). Strains belonging to this genera have a time-honored history of being used to prepare fermented foods before they were found to be an important source of various functional compounds (4). Red koji refers to solid fermenting steamed rice with red koji-mold, it is the first *Monascus*-fermented food. Meanwhile, it is an important ingredient of Asian fermented foods. Traditionally, red koji was used as the red starter to prepare several fermented foods including red-koji wine, vinegar and furu (4), now it is also be used as an alternative to statins for the treatment of dyslipidemia (5).

*Monascus*-fermented foods have become increasingly popular worldwide (3). However, to date, there are still few cases of applying this fungi genus in cheesemaking. At first, Lorrungrua et al. (6) investigated the possibility of cheese production from soymilk through a combination of *Lactobacillus casei* and *Monascus purpureus*. Yu et al. (7) mixed *M*. *purpureus* with *Penicillium* and *Geotrichum* molds for the ripening of surface-ripened cheese. Wu et al. (8) reported a surface-ripened cheese using *Monascus* fermentation broth as inoculum.

As far as we know, there is no information available on the development of internal mold-ripened cheese using red koji powder as the sole mold starter, which deserves interest in terms of either its unique creative or potential market value. In this study, we successfully developed a novel internal *Monascus*-ripened variety, called it Red-Veined Cheese. Its unique technological feature is represented by the sandwich inoculation method of red koji powder during molding.

We aim was to present the details of the manufacturing technology of Red-Veined Cheese, followed by a demonstration of the character portrait of the cheese, mainly focusing on the gross compositional and microbiological status, biochemistry changes, and volatile profile during ripening. The information obtained from this study, can not only contribute to the biochemical knowledge of *Monascus*-fermented cheese but also help to further improve the quality of this new cheese variety.

## Materials and Methods

### Materials

Raw whole cow's milk was from Bright Dairy & Food Co., Ltd. (Shanghai, China). Marzyme® 150 MG rennet was supplied by DuPont Danisco (Shanghai, China). Plate count agar and YGC medium were purchased from Qingdao Rishui Biological Technology Co., Ltd. (Qingdao, China). All other chemicals were of analytical grade and purchased from Sigma-Aldrich (Shanghai, China). *Monascus purpureus* GL-1 (CGMCC No.7603) is stored in China General Microbiological Culture Collection Center (Beijing, China). Flora Danica, a freeze-dried commercial lactic bacteria starter, containing strains *Lactococcu*s *lactis* ssp. *cremoris, Lactococcus lactis* ssp. *lactis, Lactococcus lactis* ssp. *diacetylactis* and *Leuconostoc mesenteroides* spp. *cremoris*, was provided by Chr. Hansen (Denmark).

### Preparation of Red Koji Powder

*M*. *purpureus* GL-1 was used to prepare red koji powder according to the solid-state fermentation method (9) using rice as the substrate. Experiments were conducted in 250 ml Erlenmeyer flask containing 5 g substrate (powdered rice). The substrate was moistened with salt solution and distilled water in such a way as to obtain Wnal moisture content of 60%. After thorough mixing, the wet substrates were autoclaved at 121°C for 20 min and cooled to room temperature. *M*. *purpureus* GL-1 was incubated at 30°C for 7 days and then grinded under sterile conditions. Red koji powder obtained (1.8 × 10^5^ spores/g) was used as the red starter.

### Cheese-Making and Sampling

**Figure 1:**
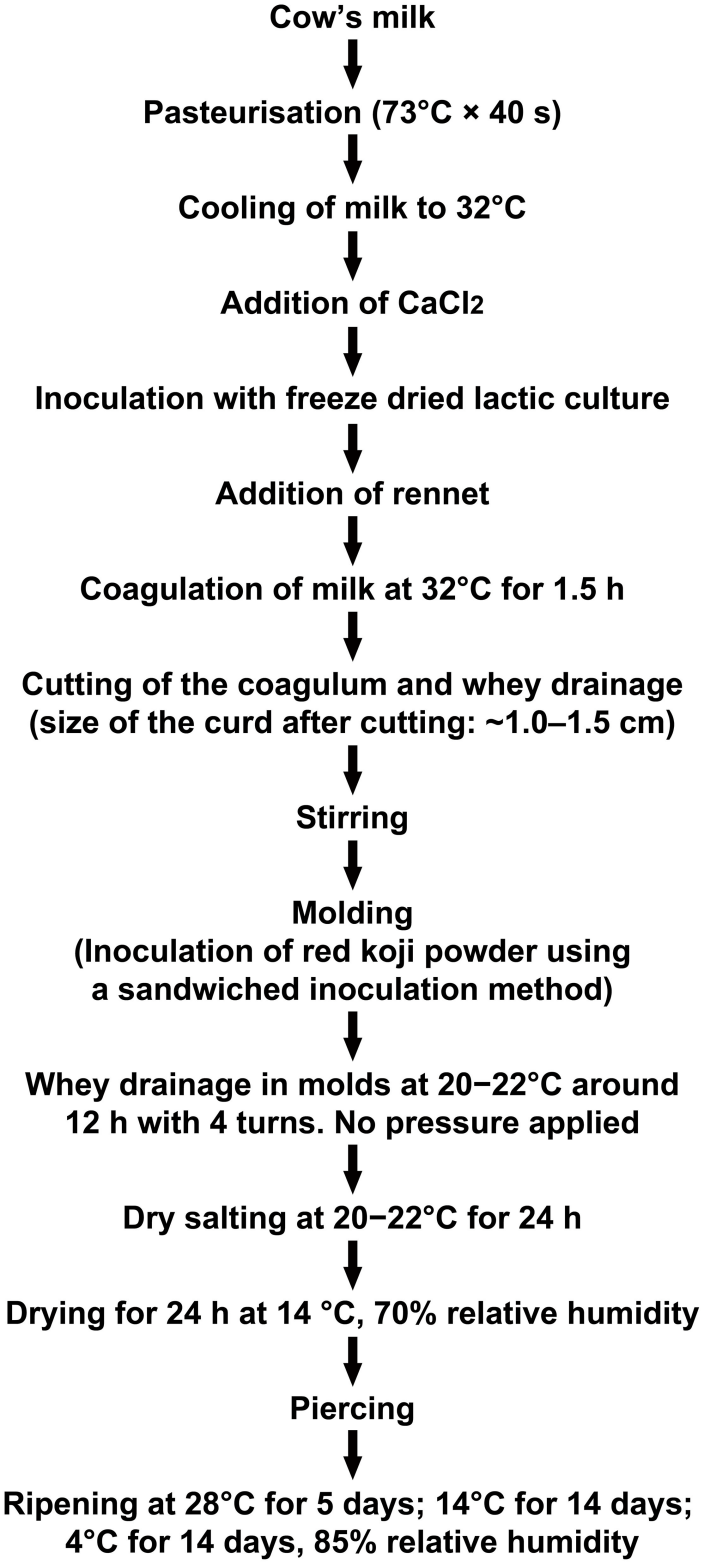
Flow diagram for the production of Red-Veined Cheese.

Seven replicas of the experimental cheeses were manufactured from ~30 liters of whole milk which was supplied by a farm of the Bright Dairy and Food Co., Ltd. according to the procedure described in Figure 1. The milk was pasteurized at 73°C for 40 s. Then, calcium chloride solution (final concentration of 9 g/100 mL of milk) and Flora Danica starter (3 g/100 L, Danisco Co. Ltd.) was added to milk at 32°C for the pre-maturation. After ~30 min (acidification up to 20°D), Marzyme® 150 MG rennet was added at a ratio of 4 g/100 L of milk. After 1.5 h, the curd was cut into hazelnut size and was stirred at ~32°C for 20 min. Then 0.2 g of red koji powder was added to each mold (diameter 11 cm, height 11 cm), more concretely: (1) transferring a layer of curd (~2.0 cm thick) to the cylindrical mold; (2) 0.05 g red koji powder was paved evenly on the surface of the curd layer, and a distance of ~1 cm from the edge of the mold was maintained; (3) transferring another layer of curd to the mold; (4) repeating step (2) and (3) until the mold was almost filled up; (5) adding the final layer of curd to encapsulate the whole cheese. After this time, the curd stayed in the molds without pressure for 12 h at 20–22°C, then was dry salted on the surface, dried in a sterile incubator and aged according to the process flow diagram shown in Figure 1. Each sampling point was made up of one of the whole replica (350 g on average) mashed after peeling and stored at −20°C before analysis. Seven replicas without inoculation of red koji powder served as the control. Strict hygienic conditions were maintained throughout the entire cheesemaking process.

### Physicochemical Parameter Analysis

Total solids, protein, fat, salt and pH were determined according to the method described by (10). All determinations were made in duplicate.

### Microbiological Analysis

Ten grams of cheese were, aseptically sampled, mixed in 90 mL of sterile 0.9% (w/v) NaCl solution. Serial decimal dilutions were prepared in the same diluent. Total mesophilic bacteria (TMB) count on PCA, incubated at 30°C for 3 days. *M*.*purpureus* GL-1 counts were enumerated on potato dextrose agar (PDA) after incubation at 25°C for 5 days.

### Nitrogen Fraction Analysis

Water-soluble nitrogen (WSN), 12% trichloroacetic acid-soluble nitrogen (TCA-SN) and 5% phosphotungstic acid-soluble nitrogen (PTA-SN) of cheeses were determined as described by Bütikofer et al. (11). Free amino acids (FAA) were analyzed according to the method of Yu et al. (7). All analyses were performed in duplicate.

### Analysis of Free Fatty Acids

Free fatty acids (FFAs) of cheese samples were extracted and esterified to methyl esters according to the method described by Felicio et al. (12). The fatty acid methyl esters were quantified by gas chromatography using an Agilent model 7890A-5975C gas chromatograph (Santa Clara, CA, USA) equipped with a flame ionization detector (FID), split injection (1:100). The column used was DB-5 (30 m × 0.25 mm × 0.25 μm i.d). The initial column temperature was programmed to 70°C for 5 min, then rising to 200°C at 25°C/min followed by an increase to 240°C at 2°C/min, then up to 300°C at 20°C/min and remaining at this temperature for 7 min. The carrier gas was helium at a flow rate of 1 mL/min. The split was 1/5, the injection volume 1 μL. The quantification of the methylated samples was made by comparison of their retention times with those of the respective standards (Sigma-Aldrich). The result was expressed as mg FFA/100 g of cheese. All analyses were conducted in duplicate.

### HS-SPME Gas Chromatography Mass Spectrometry

Volatile compound extraction was carried out using 5 g of grated cheese mixed with 8 mL NaH2PO4 solution (25%, w/v) in a 20 mL glass vial sealed with a screw cap with a PTFE-silicon septum according to the method of Mei et al. (13) with modification. Vials were then incubated at 50°C for 45 min. During the first 15 min of incubation, volatiles were allowed to be collected in the headspace and then extracted during the following 30 min using a 50/30 μm divinylbenzene/carboxen/polydimethylsiloxane (DVB/CAR/PDMS) fiber. The fiber was kept for 4 min in the injector at 260°C for desorption of volatiles then analyses were performed on a 7890A-5975C GC-MS system (Agilent Technologies, Santa Clara, CA, USA) equipped with a DB-WAX capillary column (30 m × 0.25 mm × 0.25 μm) (Agilent J &W Scientific, Folsom, CA). The oven temperature was held at 40°C for 5 min, then programmed to 250°C at 5°C/min and held at 250°C for 5 min. Helium was used as a carrier gas at a flow rate of 1 mL/min. The MS temperatures adopted were as follows: interface 280°C, source 230°C, quadrupole 150°C; the acquisition was performed in electron impact mode at 70 eV with 0.7 scans per second and the mass range used was m/z 20–400. Compounds identification was performed by comparison of mass spectra with the data from the NIST 2011 library. Linear retention indexes were also compared with bibliographic data (14). All samples were analyzed in duplicate.

### Sensory Analysis

Sensory evaluation of the cheese samples (day 5, 19, and 33 of ripening) and commercial blue cheese (president brand) was carried out according to a previous study (15) with modification. Twelve assessors were recruited and trained. Panelists gave a score between 0 (no perception) and 10 (strong perception) for each attribute, on a structured scale. Cubes of cheese core (roughly 1.5 cm^3^) were used to test for creamy, acidity, odor, salty, bitter and mushroom.

### Statistical Analysis

Means of each group were compared by one-way analysis of variance (ANOVA) followed by Tukey's test (*p* < 0.05). Comparisons of groups used the unpaired two-tailed *t*-test at a threshold of 5%. The relationship between variables in volatiles was assessed by the principal component analysis (PCA). Correlation between variables was determined by using the Pearson correlation coefficient with a *p*-value < 0.05 considered as significant. Statistics were run using SPSS version 22 software (SPSS, Inc., Chicago, IL).

## Results and Discussion

### Physicochemical and Microbial Parameters

Cheese physicochemical properties and microbial data during 33 days of ripening are summarized in Table 1. The total solids (TS) of the experimental cheese increased (*p* < 0.001) throughout ripening, finally reaching 56.7 ± 0.4 g/100 g of cheese, which was higher than those reported for Blue Cheese (16), blue-type cheeses (17) and camembert cheese (18).

**Table 1:** Changes in physicochemical and microbial parameters during cheese ripening.

**Fraction**	**Cheeses**	**Day 0**	**Day 1**	**Day 3**	**Day 5**	**Day 12**	**Day 19**	**Day 33**
Total solids (%)	EG	50.94 ± 0.33^cA^	54.57 ± 0.84^bA^	54.95 ± 0.17^bB^	54.52 ± 0.33^bB^	55.33 ± 0.22^bB^	54.50 ± 0.20^bA^	56.66 ± 0.34^aA^
	CG	51.31 ± 1.74^cA^	54.44 ± 0.41^bA^	55.74 ± 0.48^bA^	55.38 ± 0.40^bA^	55.90 ± 0.49^bA^	54.92 ± 1.67^bA^	56.83 ± 0.75^aA^
Protein (% TS)	EG	37.82 ± 0.18^aA^	37.47 ± 0.08^aA^	37.50 ± 0.66^aA^	37.04 ± 0.25^aB^	37.48 ± 0.16^aA^	37.69 ± 0.17^aA^	37.78 ± 0.24^aA^
	CG	37.24 ± 0.61^aA^	37.09 ± 1.07^aA^	37.31 ± 0.32^aA^	37.44 ± 0.16^aA^	37.09 ± 0.48^aA^	37.58 ± 0.74^aA^	37.28 ± 0.22^aB^
Fat (% TS)	EG	46.92 ± 2.78^aA^	46.00 ± 3.11^aA^	46.13 ± 1.93^aA^	45.30 ± 1.56^aA^	45.54 ± 0.77^aA^	46.24 ± 1.82^aA^	45.76 ± 2.52^aA^
	CG	47.16 ± 1.10^aA^	47.02 ± 1.56^aA^	45.93 ± 0.76^aA^	46.86 ± 0.64^aA^	46.69 ± 2.28^aA^	48.07 ± 1.29^aA^	47.87 ± 0.75^aA^
pH	EG	4.88 ± 0.04^cB^	4.96 ± 0.05^cB^	5.02 ± 0.01^bcB^	5.12 ± 0.01^abA^	5.15 ± 0.02^abA^	5.22 ± 0.06^aA^	5.23 ± 0.03^aA^
	CG	4.95 ± 0.03^cA^	5.02 ± 0.01^bcA^	5.06 ± 0.03^abcA^	5.11 ± 0.02^abA^	5.16 ± 0.07^abA^	5.17 ± 0.01^aA^	5.18 ± 0.04^aB^
NaCl (% TS)	EG	1.56 ± 0.04^dA^	1.92 ± 0.06^cdA^	2.02 ± 0.09^bcA^	2.19 ± 0.08^abcA^	2.42 ± 0.26^abA^	2.42 ± 0.01^abA^	2.58 ± 0.02^aA^
	CG	1.47 ± 0.03^fB^	1.75 ± 0.06^efB^	2.00 ± 0.01^deA^	2.12 ± 0.10^cdA^	2.31 ± 0.04^bcA^	2.53 ± 0.13^abA^	2.63 ± 0.07^aA^
TMB (log CFU/g)	EG	8.42 ± 0.07^aA^	8.06 ± 0.01^bA^	7.75 ± 0.13^bA^	7.90 ± 0.11^bA^	7.41 ± 0.09^cA^	7.34 ± 0.10^cA^	6.74 ± 0.02^dA^
	CG	8.39 ± 0.12^aA^	8.02 ± 0.09^bA^	7.84 ± 0.05^bA^	7.89 ± 0.06^bA^	7.45 ± 0.06^cA^	6.35 ± 0.10^dB^	5.77 ± 0.01^eB^
MD (log CFU/g)	EG	2.06 ± 0.03^c^	2.08 ± 0.06^c^	2.19 ± 0.1^6c^	4.99 ± 0.01^a^	4.75 ± 0.14^ab^	4.66 ± 0.08^ab^	4.32 ± 0.21^b^
	CG	ND	ND	ND	ND	ND	ND	ND

*Results are indicated as Means ± SD with different superscript lowercase letters were significantly different among ripening periods within the rows each cell (p < 0.05); Means ± SD with different superscript capital letters were significantly different among cheese samples within the column of the same ripening period (p < 0.05). TS, total solids; TMB, total mesophilic bacteria; MD, Monascus density; EG, experimental group; CG, control group; ND, not detected*.

Protein and fat showed no significant changes (*p* > 0.05) during maturation. The protein value (around 37.8 g/100 g TS) was in the range of data reported for other internal mold-ripened cheeses (16, 17, 19, 20) and almost overlapping levels measured in Danablu (37.4 g/100 g TS) and Stilton (37.9 g/100 g TS) (16). The fat level (45.8 g/100 g TS on average) was close to that of camembert-type cheese (18), but lower than in blue-veined varieties (16, 17).

As observed in other internal mold-ripened cheeses (19–21), pH neutralization process also occurred during the ripening of cheese samples. The pH value increased steadily over the course of maturation, this is mainly due to lactic acid consumption by the mold and release of ammonia and amines associated with deamination and decarboxylation of free amino acids (22). The pH value (5.22) at the end of ripening was close to that reported in Danablu and Huntsman varieties (pH = 5.3) and lower than levels recorded in other blue-veined cheeses (16, 17).

NaCl concentration increased progressively in the core part may result from the slow diffusion of salt from the surface to the center of the paste (23). The cheese sample aged 33 days showed a salt concentration (2.58 g/100 g TS) and can be classified as high-sodium cheese (24), but the value is less than halved in comparison with values reported for blue cheeses in the literature (16, 17).

The total mesophilic bacteria (TMB) count decreased from 8.42 log CFU/g of cheese on day 0–6.74 log CFU/g of cheese on day 33. A decreasing trend was similarly observed in the control group. However, after day 19, the number of TAMB was approximately an order of magnitude lower than that observed in the experimental cheese (Table 1).

The rapid increase of *Monascus* density within the first 5 days was due, firstly, to the conditions used (28°C, 85% relative humidity) which favors *Monascus* growth, and secondly, to the large number of voids distributed internally which offered enough space for mycelium growth. On the other hand, needle puncture also boosted oxygen supply and can help to distribute the mold spores throughout the paste. The *Monascus* density increased from 2.06 log CFU/g of cheese on day 0 up to 4.99 log units on day 5 (Table 1). After day 3, red veins can be visually observed inside the paste.

### Proteolysis

Evolution of different nitrogen fractions during the maturation of Red-Veined Cheese is shown in Table 2. Water-soluble nitrogen (WSN) is an important indicator to evaluate the primary proteolysis of cheese (19). Its value increased from 6.96% (on TN) at day 0–9.67% (on TN) at day 33 and remained at a higher level throughout the ripening process as compared with the control without *Monascus* inoculation.

**Table 2:** Changes of nitrogenous fractions during cheese ripening.

**Fraction**	**Cheeses**	**Day 0**	**Day 1**	**Day 3**	**Day 5**	**Day 12**	**Day 19**	**Day 33**
WSN (% on TN)	EG	6.96 ± 0.44^bA^	7.49 ± 0.03^bA^	8.03 ± 0.64^abA^	8.69 ± 0.16^abA^	9.00 ± 0.03^abA^	9.62 ± 0.03^aA^	9.67 ± 0.59^aA^
	CG	6.86 ± 0.10^aA^	7.11 ± 0.42^aA^	7.21 ± 0.17^aB^	8.30 ± 0.03^aB^	8.55 ± 0.00^aB^	8.57 ± 0.09^aB^	8.56 ± 1.13^aA^
TCA-SN (% on TN)	EG	4.14 ± 0.13^bB^	4.90 ± 0.82^abA^	5.51 ± 0.11^abA^	5.94 ± 0.49^abA^	6.21 ± 0.02^abA^	7.14 ± 0.46^aA^	7.14 ± 0.87^aA^
	CG	4.50 ± 0.21^aA^	4.29 ± 0.42^aA^	4.39 ± 0.19^aB^	5.50 ± 0.50^aA^	5.71 ± 0.56^aA^	5.79 ± 0.45^aB^	5.63 ± 0.04^aB^
PTA-SN (% on TN)	EG	0.81 ± 0.02^aA^	1.28 ± 0.30^aA^	1.83 ± 0.45^aA^	3.09 ± 0.60^aA^	3.73 ± 1.77^aA^	5.03 ± 1.83^aA^	4.94 ± 0.23^aA^
	CG	0.53 ± 0.03^aB^	1.00 ± 0.19^aA^	1.14 ± 0.65^aA^	1.72 ± 0.35^aB^	1.65 ± 0.43^aB^	2.37 ± 0.94^aB^	2.70 ± 0.18^aB^
TFAA (mg/g TS)	EG	0.98 ± 0.29^aA^	0.99 ± 0.12^aA^	1.14 ± 0.16^aA^	1.18 ± 0.41^aA^	1.26 ± 0.26^aA^	1.31 ± 0.16^aA^	1.38 ± 0.04^aA^
	CG	0.97 ± 0.17^aA^	1.01 ± 0.02^aA^	1.03 ± 0.23^aA^	1.05 ± 0.31^aA^	1.18 ± 0.19^aA^	1.28 ± 0.09^aA^	1.33 ± 0.12^aA^

*Results are indicated as Means ± SD with different superscript lowercase letters were significantly different among ripening periods within the rows each cell (p < 0.05); Means ± SD with different superscript capital letters were significantly different among cheese samples within the column of the same ripening period (p < 0.05). WSN, water-soluble nitrogen; TCA-SN, nitrogen soluble in 12% TCA; PTA-SN, nitrogen soluble in 5% phosphotungstic acid; TN, total nitrogen; TFAA, total free amino acids; TS, total solids; EG, experimental group; CG, control group*.

TCA-SN fraction, as an index of secondary proteolysis, contains short peptides and FAAs (25). After 12 days of ripening, TCA-SN further increased in the experimental cheese. At day 33, the value has increased by 70% of its initial value. TCA-SN development was found positively correlated with the *Monascus* density (*r* = 0.798, *p* < 0.05), indicating the contribution of *Monascus* fermentation in the paste to secondary proteolysis of the cheese.

As shown in the table, a significant accumulation of PTA-SN fraction throughout the maturation [from 0.81 ± 0.02% (on TN) at day 0 to 4.94 ± 0.23% (on TN) at day 33] was accompanied by a dramatic enhance in the proportion of PTA-SN to WSN (from 11.6 to 51.1%). Meanwhile, the ratio of PTA-SN (expressed as % TCA-SN) also increased from 19.6% at the beginning to 69.2% at the end of the experiment, demonstrating balance shifting between primary and secondary proteolysis in which the latter gradually predominated. At the end of maturation, PTA-SN accumulation increased 1.8-fold compared to the control.

As expected, the experimental cheese displayed enhanced proteolysis compared with the control. Since the evolution of nitrogen fractions during maturation was mainly attributed to the microbial peptidase activity. The increase in the rate of proteolysis could be probably related to the metabolic activity of *M*. *purpureus* GL-1. Additionally, a relatively higher load of lactic acid bacteria in the experimental cheese at the later stage of ripening may also help to promote proteolysis of the cheese.

**Figure 2:**
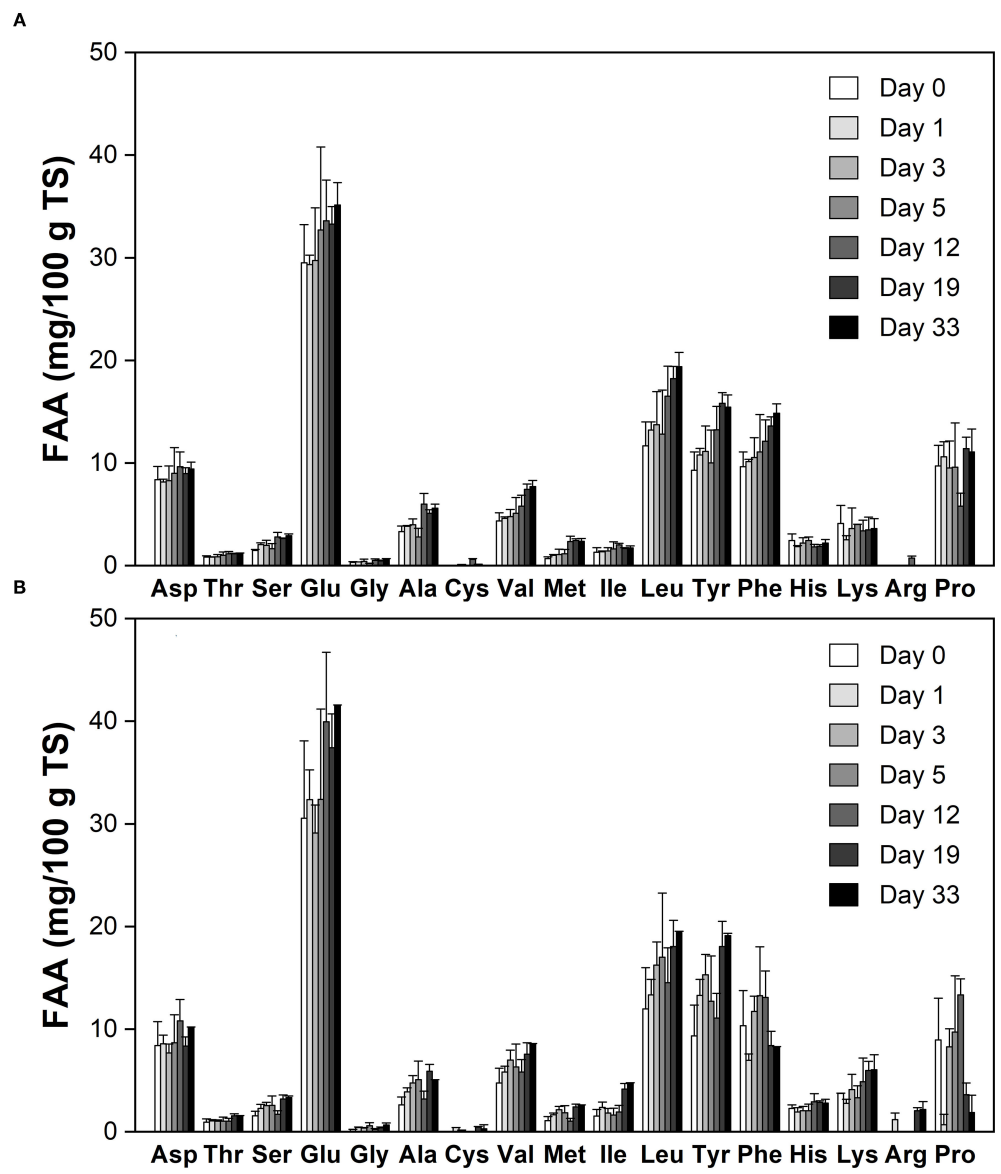
Free amino acid (FAA) profiles during cheese ripening. **(A)** The control without inoculation of red koji powder; **(B)** Red-Veined Cheese. Error bars indicate standard deviations from the mean.

Figure 2 shows the free amino acid (FAA) profiles during cheese ripening. Most of FAAs displayed increasing trends in the experimental group (Figure 2B). Phe and Pro were decreased with time, possibly because they were consumed by the mold or degraded to amines and other FAAs through the decarboxylation and deamination (26). At day 33, concentrations of Glu, Lys, His, Ser, Tyr, Ile, and Arg were significantly higher (*p* < 0.01) than that of the control (Figure 2A). The most abundant FAAs at all stages of ripening were Glu, Leu and Tyr, which together accounted for nearly 60% of the total FAA in the experimental cheese, whereas their percent contribution in blue cheese is only ~40% (27). Thr, Ser, Gly, Cys, Val, Met, His, Arg, and Pro make up 23% of the total FAA content in the ripened experimental cheese, while this ratio is over 40% in blue-veined varieties (27). This indicates that there is significant discrimination between Red-Veined Cheese and traditional internal mold-ripened cheese in the FAA profile.

The total FAA remained at a low level until the end of ripening (Table 2), indicating that one month's maturity might have been too short compared with that of blue-veined varieties (21, 28, 29) to promote great differences in proteolysis degree.

### Lipolysis

Changes in fatty acid composition of cheese during 33 days of ripening are summarized in Table 3. The total FFA content of the experimental cheese nearly doubled throughout the maturation, indicating that the cheese went through substantial lipolysis during ripening. At day 33, palmitic acid (C_16 : 0_) is the most abundant in the ripened experimental cheese, followed by oleic acid (C_18 : 1_), accounting together for 52.3% of total free fatty acids (TFFA). Compared with the control, long-chain saturated fatty acids (LCSFA) decreased by 7%, while long-chain unsaturated fatty acids (LCUSFA) and Short-chain fatty acids (SCFA) increased by 45 and 12%, respectively.

**Table 3:** Changes of free fatty acids (FFA) content (mg/100 g of cheese) in cheeses during ripening.

**Fraction**	**Cheeses**	**Day 0**	**Day 1**	**Day 3**	**Day 5**	**Day 12**	**Day 19**	**Day 33**
C_6 : 0_	EG	2.75 ± 0.05^aA^	3.45 ± 2.08^aA^	3.60 ± 0.16^aA^	4.38 ± 1.05^aA^	2.69 ± 0.11^aA^	1.75 ± 0.22^aB^	1.64 ± 0.30^aB^
	CG	2.73 ± 0.04^bA^	3.21 ± 0.82^abA^	2.84 ± 0.32^bB^	3.74 ± 0.03^abA^	3.18 ± 0.78^abA^	4.23 ± 0.07^abA^	5.00 ± 0.52^aA^
C_8 : 0_	EG	2.58 ± 0.36^aB^	3.07 ± 0.52^aA^	2.99 ± 0.23^aA^	3.12 ± 1.50^aA^	3.11 ± 0.32^aA^	5.47 ± 0.66^aB^	3.79 ± 0.71^aB^
	CG	3.00 ± 0.13^aA^	3.68 ± 0.53^aA^	4.82 ± 2.40^aA^	4.23 ± 0.03^aA^	3.59 ± 0.81^aA^	6.64 ± 0.10^aA^	5.26 ± 0.56^aA^
C_10 : 0_	EG	8.53 ± 2.82^bB^	16.15 ± 5.83^bA^	11.67 ± 0.87^bA^	17.21 ± 2.61^bA^	11.87 ± 1.48^bA^	58.46 ± 2.10^aA^	66.00 ± 17.46^aA^
	CG	11.38 ± 0.86^bA^	14.24 ± 0.64^bA^	11.21 ± 2.75^bA^	15.35 ± 0.54^bA^	13.79 ± 2.79^bA^	48.80 ± 7.35^aB^	53.58 ± 9.36^aA^
SCFA	EG	13.87 ± 2.52^bB^	22.68 ± 8.43^bA^	18.26 ± 1.26^bA^	24.71 ± 2.16^bA^	17.66 ± 1.91^bA^	65.68 ± 2.54^aA^	71.44 ± 17.86^aA^
	CG	17.11 ± 1.04^bA^	21.12 ± 1.98^bA^	18.87 ± 0.03^bA^	23.32 ± 0.60^bA^	20.56 ± 4.37^bA^	59.67 ± 7.32^aA^	63.84 ± 10.44^aA^
C_12 : 0_	EG	15.53 ± 7.44^cA^	28.21 ± 7.19^cA^	20.71 ± 1.50^cB^	27.58 ± 2.45^cA^	20.58 ± 2.56^cA^	171.06 ± 9.94^bA^	199.29 ± 9.32^aA^
	CG	20.41 ± 1.57^cA^	24.98 ± 0.62^cA^	25.20 ± 0.56^cA^	25.43 ± 1.14^cA^	24.09 ± 4.14^cA^	86.90 ± 15.61^bB^	132.03 ± 8.29^aB^
C_14 : 0_	EG	4.54 ± 0.11^cA^	5.91 ± 0.69^bcA^	4.88 ± 0.60^bcB^	5.46 ± 0.48^bcA^	4.45 ± 0.43^cA^	14.16 ± 3.61^aA^	12.31 ± 3.23^abA^
	CG	4.65 ± 0.09^aA^	5.43 ± 0.27^aA^	5.67 ± 0.30^aA^	5.41 ± 0.34^aA^	5.28 ± 0.79^aA^	5.45 ± 1.01^aB^	6.85 ± 0.72^aB^
C_16 : 0_	EG	342.57 ± 24.01^aA^	343.68 ± 9.17^aA^	359.77 ± 31.94^aA^	368.17 ± 6.79^aA^	349.66 ± 31.85^aA^	392.71 ± 25.69^aA^	404.20 ± 32.51^aB^
	CG	341.85 ± 60.73^aA^	355.28 ± 54.12^aA^	361.34 ± 95.42^aA^	367.21 ± 2.21^aA^	391.35 ± 41.01^aA^	445.12 ± 59.39^aA^	480.58 ± 70.22^aA^
C_18 : 0_	EG	148.86 ± 6.13^aA^	157.09 ± 18.42^aA^	137.09 ± 10.17^aB^	148.13 ± 0.58^aA^	135.55 ± 10.72^aB^	125.67 ± 25.06^aB^	134.66 ± 28.83^aB^
	CG	139.09 ± 11.36^aA^	157.02 ± 4.93^aA^	158.02 ± 10.09^aA^	144.20 ± 4.94^aA^	153.52 ± 9.90^aA^	165.26 ± 22.65^aA^	184.96 ± 30.33^aA^
LCSFA	EG	511.51 ± 25.43^bA^	534.88 ± 17.14^bA^	522.45 ± 19.67^bA^	549.35 ± 3.29^bA^	510.24 ± 45.56^bA^	703.61 ± 12.91^aA^	750.45 ± 9.78^aA^
	CG	506.00 ± 73.76^bA^	542.71 ± 48.30^abA^	550.23 ± 104.66^abA^	542.24 ± 4.21^abB^	574.25 ± 55.85^abA^	702.72 ± 65.42^abA^	804.42 ± 91.54^aA^
C_16 : 1_	EG	10.34 ± 6.12^bA^	17.98 ± 2.43^bA^	14.93 ± 1.42^bA^	16.77 ± 1.77^bA^	14.37 ± 1.41^bA^	121.79 ± 39.88^aA^	123.52 ± 30.54^aA^
	CG	14.42 ± 0.88^cA^	17.08 ± 0.80^cA^	16.47 ± 1.15^cA^	16.40 ± 1.08^cA^	16.69 ± 2.34^cA^	61.33 ± 4.88^bB^	73.95 ± 3.18^aB^
C_18 : 1_	EG	267.22 ± 27.85^cA^	281.20 ± 8.78^bcA^	261.83 ± 7.92^cB^	282.72 ± 12.26^bcA^	346.24 ± 20.88^abA^	356.41 ± 21.85^aA^	363.70 ± 19.52^aA^
	CG	252.74 ± 26.51^bA^	273.95 ± 6.87^abA^	280.07 ± 9.97^abA^	257.29 ± 8.53^bB^	271.37 ± 25.72^abB^	281.17 ± 6.88^abB^	333.35 ± 25.91^aB^
C_18 : 2_	EG	17.26 ± 9.14^bA^	25.83 ± 1.85^bA^	22.56 ± 0.54^bA^	24.83 ± 0.47^bA^	22.23 ± 0.97^bA^	146.68 ± 42.53^aA^	160.08 ± 51.79^aA^
	CG	22.77 ± 1.14^aA^	24.96 ± 1.40^aA^	21.85 ± 4.81^aA^	24.32 ± 1.23^aA^	24.50 ± 2.30^aA^	31.52 ± 1.65^aB^	38.80 ± 14.16^aB^
LCUSFA	EG	294.82 ± 12.59^bA^	325.02 ± 13.06^bA^	299.32 ± 9.88^bB^	324.32 ± 14.49^bA^	382.85 ± 23.26^bA^	624.87 ± 60.56^aA^	647.30 ± 62.82^aA^
	CG	289.94 ± 28.54^cA^	315.99 ± 9.07^bcA^	318.38 ± 15.93^bcA^	298.01 ± 10.84^cB^	312.57 ± 30.35^bcB^	374.02 ± 10.12^abB^	446.09 ± 14.93^aB^
Total FFA	EG	820.20 ± 15.36^bA^	882.58 ± 38.63^bA^	840.04 ± 8.52^bA^	898.38 ± 13.36^bA^	910.75 ± 70.73^bA^	1,394.17 ± 76.01^aA^	1,469.19 ± 70.90^aA^
	CG	813.04 ± 103.33^bA^	879.82 ± 41.22^bA^	887.48 ± 120.62^bA^	863.57 ± 15.65^bB^	907.38 ± 90.57^bA^	1,136.41 ± 68.22^abB^	1,314.35 ± 96.02^aB^

*Results are indicated as Means ± SD with different superscript lowercase letters were significantly different among ripening periods within the rows each cell (p < 0.05); Means ± SD with different superscript capital letters were significantly different among cheese samples within the column of the same ripening period (p < 0.05). FFA, free fatty acids; SCFA, short-chain fatty acids (C_6 : 0_, C_8 : 0_, C_10 : 0_); LCSFA, long-chain saturated fatty acids (C_12 : 0_, C_14 : 0_, C_16 : 0_ and C_18 : 0_); LCUSFA, long-chain unsaturated fatty acids (C_16 : 1_, C_18 : 1_, C_18 : 2_); EG, experimental group; CG, control group*.

**Figure 3:**
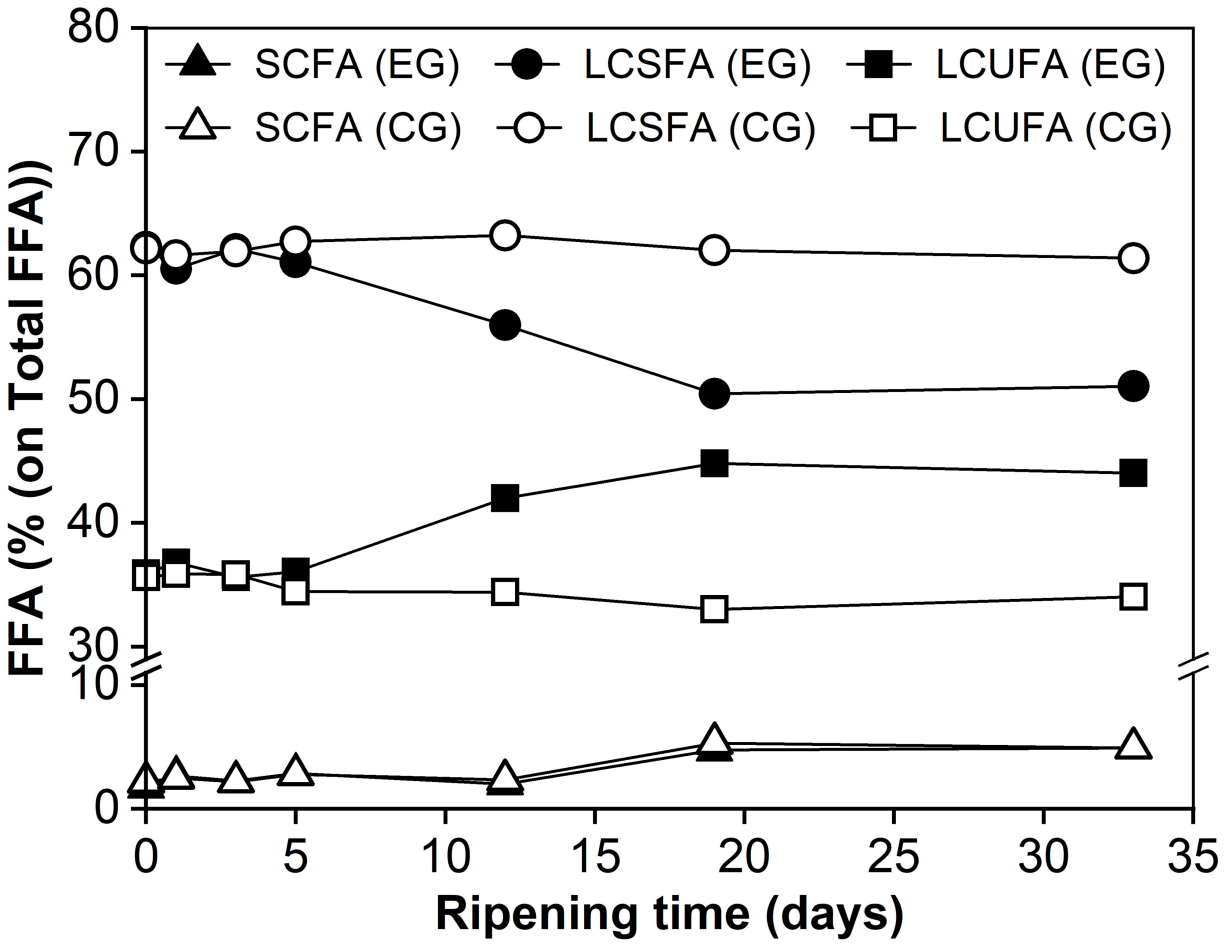
The relative abundance of free fatty acids (FFAs) categorized based on chain length and saturation degree during cheese ripening. SCFA, short-chain fatty acids (C_6 : 0_, C_8 : 0_, C_10 : 0_); LCSFA, long-chain saturated fatty acids (C_12 : 0_, C_14 : 0_, C_16 : 0_ and C_18 : 0_); LCUFA, long-chain unsaturated fatty acids (C_16 : 1_, C_18 : 1_, C_18 : 2_); EG, experimental group; CG, control group without inoculation of red koji powder.

Changes in the relative abundance of free fatty acids (FFAs) with different chain lengths and saturations are shown in Figure 3. It can be seen that the mass fractions did not change for FFAs with varying chain lengths within the first 5 days. Over the remainder of the maturation period, the fraction of LCSFA decreased from 62 to 51% whereas the proportion of LCUFA increased from 36 to 44%. In contrast, similar changes were not observed in the control group. This difference could be due at least in part to the metabolic activity of *M. purpureus* GL-1. For the SCFA fraction, its proportion increased progressively over time, with similar trends seen in both groups. At day 33, SCFA only represented ~5% of Total FFA in the experimental cheese, which may be due to their consumption by the mold, or more likely, converted to other volatile compounds to contribute to cheese flavor (30).

The Total FFA content reached a final amount of 1469.19 mg/100 g of cheese (Table 3), which was higher than those of Camembert (681–5,066 mg/kg of cheese) and Brie (2,678 mg/kg of cheese), but lower than those reported for blue cheese (over 26 g/kg of cheese). The latter might be because the maturation takes a much shorter time than blue cheese. Additionally, the percentage of unsaturated fatty acids (44%) was higher in the ripened experimental cheese than in traditional internal mold-ripened and surface-ripened cheeses (30). At the late stage of ripening, the total FFA content showed a significantly higher level (*p* < 0.05) in the experimental group, suggesting that internal *Monascus*-fermentation did promote cheese lipolysis.

### Volatile Compounds

Table 4 summarizes 63 volatile compounds identified during the ripening of Red-Veined Cheese by the HS-SPM-GC-MS method, including 12 Ketones, 14 alcohols, 15 acids, 13 esters and 5 aldehydes, 3 lactones and 1 phenol.

**Table 4:** Volatile compounds identified in Red-Veined Cheese by HS-SPME-GC-MS during the different ripening stages.

**Compounds**	**Code**	**LRI**	**IM**	**Day 0**	**Day 1**	**Day 3**	**Day 5**	**Day 12**	**Day 19**	**Day 33**	***P***
***Ketones***											
Acetone	A1	856	MS, PI	147.67 ± 32.06^b^	141.43 ± 4.69^b^	144.32 ± 3.86^b^	168.39 ± 16.80^ab^	199.98 ± 34.49^ab^	239.88 ± 2.11^a^	198.69 ± 11.46^ab^	^*^
2,3-Butanedione	A2	957	MS, PI	93.31 ± 5.11^a^	25.90 ± 1.40^b^	11.94 ± 0.68^c^	ND	ND	ND	ND	^***^
2-Pentanone	A3	958	MS, PI	ND	ND	11.94 ± 0.68^b^	57.41 ± 6.42^ab^	74.96 ± 26.14^a^	73.75 ± 1.82^a^	66.16 ± 10.59^a^	^*^
2-Hexanone	A4	1,077	MS, PI	ND	ND	1.13 ± 0.17^b^	2.68 ± 0.35^b^	7.47 ± 0.77^b^	21.84 ± 5.39^a^	10.89 ± 0.03^b^	^**^
2-Heptanone	A5	1,202	MS, PI	6.37 ± 0.92^d^	11.37 ± 2.75^d^	22.68 ± 5.56^d^	62.78 ± 15.79^d^	309.78 ± 106.30^c^	1,597.00 ± 107.93^a^	873.30 ± 18.15^b^	^***^
2-Octanone	A6	1,292	MS, PI	ND	ND	ND	2.91 ± 0.71^d^	34.34 ± 3.44^c^	157.70 ± 5.93^a^	50.01 ± 1.16^b^	^***^
Acetoin	A7	1,294	MS	71.83 ± 9.87^b^	14.07 ± 4.83^c^	22.71 ± 2.27^c^	16.19 ± 4.29^c^	26.79 ± 6.03^bc^	170.05 ± 23.39^a^	53.61 ± 13.91b^c^	^***^
2-Nonanone	A8	1,392	MS, PI	9.88 ± 0.79^d^	14.29 ± 6.83^d^	24.97 ± 1.94^d^	46.11 ± 14.68^d^	376.19 ± 90.41^c^	2,031.11 ± 96.05^a^	1,609.96 ± 25.86^b^	^***^
8-Nonen-2-one	A9	1,435	MS, PI	ND	ND	2.89 ± 0.41^b^	7.29 ± 1.78^b^	72.72 ± 14.97^b^	296.08 ± 25.72^a^	244.52 ± 27.30^a^	^***^
2-Undecanone	A10	1,583	MS, PI	ND	ND	0.27 ± 0.09^b^	4.10 ± 0.77^b^	16.99 ± 3.96^b^	50.01 ± 0.48^a^	63.92 ± 15.53^a^	^**^
2-Dodecanone	A11	1,591	MS	ND	ND	1.83 ± 0.81^b^	5.84 ± 0.91^a^	5.58 ± 0.96^a^	4.44 ± 0.07^ab^	ND	^*^
2-Decanone	A12	1,794	MS	ND	ND	2.86 ± 0.96^c^	2.75 ± 0.52^c^	2.84 ± 0.36^c^	17.80 ± 1.30^a^	8.15 ± 0.58^b^	^***^
**Alcohols**											
Ethanol	B1	922	MS, PI	805.41 ± 145.02^a^	584.45 ± 184.41^ab^	296.96 ± 24.63^bc^	177.47 ± 10.94^c^	165.93 ± 3.56^c^	930.10 ± 40.75^a^	207.65 ± 41.46^c^	^***^
1-Butanol	B2	1,171	MS, PI	1.16 ± 0.23	ND	ND	ND	ND	ND	ND	
2-Methyl-1-butanol	B3	1,233	MS	1.43 ± 0.55	ND	ND	ND	ND	ND	ND	
3-Methyl-1-butanol	B4	1,249	MS, PI	0.99 ± 0.44^d^	1.82 ± 0.34^d^	5.46 ± 2.18^d^	63.52 ± 1.30^c^	124.02 ± 20.93^b^	111.75 ± 10.41^bc^	251.17 ± 29.41^a^	^***^
1-Pentanol	B5	1,273	MS, PI	ND	ND	ND	1.71 ± 0.82^d^	4.98 ± 0.50^c^	13.70 ± 0.54^b^	19.02 ± 0.96^a^	^***^
2-Heptanol	B6	1,337	MS	5.83 ± 0.80^c^	3.94 ± 0.81^c^	2.23 ± 1.62^c^	2.66 ± 0.89^c^	4.36 ± 0.57^c^	42.57 ± 4.37^a^	22.51 ± 0.13^b^	^***^
1-Hexanol	B7	1,364	MS, PI	13.71 ± 2.74^a^	0.96 ± 0.01^c^	1.10 ± 0.24^c^	2.23 ± 0.05^bc^	3.03 ± 1.05^bc^	11.96 ± 1.52^a^	6.51 ± 0.52^b^	^***^
2-Ethyl-1-hexanol	B8	1,488	MS, PI	ND	ND	ND	ND	ND	ND	7.49 ± 0.36	
2-Decanol	B9	1,498	MS	ND	ND	ND	ND	ND	3.71 ± 0.71^a^	3.68 ± 0.05^a^	NS
2-Nonanol	B10	1,518	MS, PI	8.27 ± 0.70^c^	6.88 ± 0.48^c^	3.71 ± 0.81^c^	3.00 ± 0.93^c^	4.78 ± 1.39^c^	52.15 ± 4.52^b^	64.76 ± 2.53^a^	^***^
1-Octanol	B11	1,553	MS	21.24 ± 1.15^b^	21.23 ± 0.99^b^	9.76 ± 2.22^c^	8.21 ± 2.31^c^	13.28 ± 0.20^c^	36.05 ± 1.87^a^	27.32 ± 0.88^b^	^***^
1-Nonanol	B12	1,651	MS	ND	ND	ND	ND	ND	41.14 ± 12.96^a^	44.73 ± 11.33^a^	NS
Benzyl alcohol	B13	1,849	MS	ND	ND	3.52 ± 0.33^c^	16.07 ± 4.67^bc^	44.33 ± 6.62^b^	424.18 ± 15.47^a^	416.80 ± 9.56^a^	^***^
2-Phenylethanol	B14	1,885	MS, PI	ND	ND	58.94 ± 10.96^c^	430.95 ± 152.24^c^	1,212.42 ± 161.00^b^	2,283.25 ± 130.19^a^	1,891.03 ± 168.79^a^	^***^
**Acids**											
Acetic acid	C1	1,437	MS, PI	369.74 ± 18.78^a^	304.18 ± 68.50^ab^	304.52 ± 44.76^ab^	175.23 ± 16.77^bc^	13.89 ± 4.11^d^	134.79 ± 20.34^cd^	160.46 ± 26.11^c^	^***^
Propanoic acid	C2	1,526	MS, PI	ND	ND	ND	ND	ND	ND	7.50 ± 0.93	
Isobutyric acid	C3	1,555	MS, PI	ND	ND	ND	ND	13.15 ± 0.02^b^	36.05 ± 1.87^a^	36.81 ± 6.00^a^	^*^
Butanoic acid	C4	1,608	MS, PI	50.26 ± 5.48^b^	43.77 ± 2.37^b^	27.56 ± 5.52^b^	26.31 ± 5.89^b^	42.21 ± 0.26^b^	109.66 ± 4.81^a^	118.32 ± 18.04^a^	^***^
2-Methylbutanoic acid	C5	1,652	MS	ND	ND	ND	ND	48.25 ± 14.09	ND	ND	
3-Methylbutanoic acid	C6	1,653	MS, PI	ND	ND	ND	4.83 ± 0.98^d^	47.06 ± 13.29^c^	527.40 ± 9.71^a^	112.53 ± 3.19^b^	^***^
Hexanoic acid	C7	1,823	MS, PI	148.70 ± 13.29^b^	144.58 ± 3.97^b^	122.05 ± 27.81^bc^	71.31 ± 0.16^bc^	42.77 ± 3.23^c^	246.78 ± 43.74^a^	251.69 ± 2.74^a^	^***^
Heptanoic acid	C8	1,967	MS, PI	ND	ND	2.97 ± 0.02^c^	2.75 ± 1.20^cd^	0.18 ± 0.06^d^	15.35 ± 0.91^a^	5.79 ± 0.18^b^	^***^
Octanoic acid	C9	2,039	MS, PI	102.23 ± 3.34^c^	120.11 ± 13.19^c^	161.68 ± 36.92^c^	189.54 ± 52.03^c^	92.98 ± 14.99^c^	628.32 ± 6.64^a^	464.68 ± 68.40^b^	^***^
Nonanoic acid	C10	2,152	MS, PI	ND	ND	ND	ND	5.78 ± 1.17	ND	ND	
Decanoic acid	C11	2,255	MS, PI	64.55 ± 10.35^bc^	62.56 ± 11.73^c^	111.05 ± 18.53^bc^	131.39 ± 31.61^b^	128.10 ± 16.84^bc^	408.44 ± 13.85^a^	395.83 ± 6.72^a^	^***^
9-Decenoic acid	C12	2,315	MS	ND	ND	ND	ND	ND	27.36 ± 1.12^a^	21.27 ± 0.02^b^	^*^
Dodecanoic acid	C13	2,466	MS	23.28 ± 8.82^b^	31.76 ± 7.17^b^	49.07 ± 26.99^b^	52.83 ± 4.53^b^	52.81 ± 7.54^b^	139.24 ± 14.24^a^	114.63 ± 7.90^a^	^***^
Tetradecanoic acid	C14	2,669	MS	4.39 ± 1.22^b^	7.84 ± 0.96^b^	55.02 ± 11.08^ab^	91.60 ± 0.03^a^	97.83 ± 52.12^a^	64.63 ± 4.19^ab^	84.44 ± 14.28^ab^	^*^
Hexadecanoic acid	C15	2,854	MS	ND	25.57 ± 1.56^d^	174.11 ± 16.43^c^	258.18 ± 29.80^b^	416.25 ± 2.52^a^	194.25 ± 0.89^c^	218.26 ± 13.51^bc^	^***^
**Esters**											
Ethyl acetate	D1	886	MS, PI	ND	ND	ND	ND	ND	5.54 ± 1.20	ND	
Ethyl butyrate	D2	1,032	MS, PI	56.34 ± 4.41^b^	47.57 ± 4.90^b^	13.34 ± 1.24^c^	12.95 ± 2.94^c^	8.47 ± 3.06^c^	100.13 ± 11.78^a^	40.22 ± 0.85^b^	^***^
Butyl acetate	D3	1,070	MS, PI	1.99 ± 1.36^a^	1.47 ± 0.03^a^	0.37 ± 0.06^a^	ND	ND	2.48 ± 1.29^a^	3.00 ± 0.01^a^	NS
Butyl acrylate	D4	1,194	MS	0.84 ± 0.14	ND	ND	ND	ND	ND	ND	
Ethyl caproate	D5	1,253	MS, PI	103.81 ± 6.18^b^	122.75 ± 1.18^b^	77.97 ± 13.27^b^	75.18 ± 17.79^b^	72.88 ± 23.28^b^	959.12 ± 327.49^a^	333.54 ± 19.38^b^	^**^
Isopentyl butyrate	D6	1,270	MS	ND	ND	ND	ND	2.67 ± 0.33^b^	26.67 ± 2.28^a^	1.49 ± 0.01^b^	^**^
Propyl hexanoate	D7	1,321	MS, PI	ND	ND	ND	ND	ND	ND	4.70 ± 0.32	
Ethyl heptanoate	D8	1,334	MS, PI	ND	ND	3.06 ± 0.45^b^	3.13 ± 0.23^b^	3.58 ± 1.67^b^	42.52 ± 4.44^a^	9.96 ± 0.76^b^	^***^
Ethyl octanoate	D9	1,438	MS, PI	23.66 ± 7.23^c^	26.10 ± 11.89^c^	69.46 ± 14.90^c^	73.24 ± 28.04^c^	87.81 ± 17.29^c^	419.21 ± 32.28^a^	264.43 ± 23.22^b^	^***^
Isopentyl hexanoate	D10	1,451	MS, PI	ND	ND	0.68 ± 0.15^d^	9.16 ± 2.05^c^	9.98 ± 0.37^c^	34.90 ± 1.97^a^	22.62 ± 0.98^b^	^***^
Ethyl nonanoate	D11	1,524	MS	ND	ND	2.20 ± 1.11^c^	2.91 ± 0.33^bc^	7.09 ± 0.42^b^	15.69 ± 1.65^a^	12.14 ± 1.10^a^	^***^
Ethyl caprate	D12	1,624	MS, PI	7.91 ± 1.24^d^	14.33 ± 1.47^d^	41.63 ± 8.56^cd^	53.41 ± 25.12^cd^	75.99 ± 12.39^c^	209.13 ± 9.60^a^	133.61 ± 3.58^b^	^***^
Ethyl laurate	D13	1,832	MS	ND	ND	7.43 ± 1.30^c^	10.02 ± 3.91^bc^	17.42 ± 0.79^b^	38.38 ± 1.12^a^	31.71 ± 1.34^a^	^***^
**Aldehydes**											
3-Methylbutanal	E1	906	MS, PI	ND	ND	0.29 ± 0.03^b^	1.49 ± 0.09^a^	ND	ND	ND	^**^
Hexanal	E2	1,077	MS	3.10 ± 0.66	ND	ND	ND	ND	ND	ND	
Nonanal	E3	1,396	MS, PI	8.01 ± 2.42^bc^	4.76 ± 2.54^c^	4.08 ± 1.11^c^	3.13 ± 0.23^c^	18.98 ± 0.10^ab^	31.05 ± 6.24^a^	31.60 ± 5.46^a^	^***^
Benzaldehyde	E4	1,501	MS, PI	ND	ND	2.41 ± 1.07^c^	41.46 ± 5.86^b^	68.19 ± 15.95^ab^	89.27 ± 2.48^a^	64.99 ± 1.48^ab^	^**^
Benzeneacetaldehyde	E5	1,613	MS	ND	1.02 ± 0.17^b^	18.30 ± 7.57^b^	20.47 ± 6.02^b^	60.59 ± 11.87^a^	17.60 ± 3.72^b^	22.67 ± 6.46^b^	^**^
Total				11.11 ± 1.76^c^	5.78 ± 2.37^c^	25.09 ± 5.36^bc^	66.56 ± 11.56^b^	147.76 ± 27.92^a^	137.92 ± 5.00^a^	119.27 ± 10.44^a^	^***^
***Lactones***											
Butyrolactone	F1	1,600	MS	ND	ND	ND	1.59 ± 0.30^b^	1.50 ± 0.11^b^	7.52 ± 1.43^a^	2.47 ± 0.05^b^	^**^
δ-Octalactone	F2	1,944	MS	2.73 ± 0.03^a^	2.54 ± 0.31^a^	3.00 ± 0.17^a^	2.69 ± 0.78^a^	2.64 ± 0.49^a^	5.32 ± 1.67^a^	3.06 ± 0.18^a^	NS
δ-Decanolactone	F3	2,171	MS	7.59 ± 1.08^d^	23.09 ± 0.60^bc^	17.59 ± 2.85^c^	14.36 ± 5.51^cd^	16.77 ± 0.75^cd^	34.93 ± 1.50^a^	31.37 ± 1.24^ab^	^***^
**Phenols**											
Phenol	G1	1,972	MS	ND	ND	ND	ND	ND	1.25 ± 0.26	ND	

* HS-SPME-GC-MS data are expressed as arbitrary units (×10^5^) of average peak area ± standard deviations for each compound. Different superscript lowercase letters in the same row denote statistical difference (p < 0.05) between samples according to the Tukey test. LRI: linear retention index using a DB-WAX column; IM, identification method; P, probability value. NS, no significant;

* P < 0.05;

** P < 0.01;

*** *P < 0.001; MS, mass spectra comparison using NIST library; PI, comparison with published LRI; ND, not detected*.

#### Ketones

Ketones consisted of 12 components. The majority being methyl ketones. At the end of ripening, the GC-MS total peak area of this chemical group accounted for 35% of all volatiles. The most abundant 2-alkanones were the ones with an odd number of carbon atoms. Among which, 2-nonanone was the most abundant compound, next is 2-heptanone, together representing ~80% of total ketones. In blue cheeses, they are characteristic flavor components which are formed during enzymatic lipolysis of FFAs (31). Although ketones was the most abundant group in the ripened experimental cheese, its percentage in neutral volatiles (38%) is significantly lower than that observed in blue cheeses (50–70%) (32, 33).

#### Alcohols

Alcohols became the second abundant flavor component after 2 weeks, accounting for more than 30% of the total volatiles. Most of them belong to primary alcohols. Secondary alcohols detected (from C3 to C9) in Red-Veined Cheese are typical flavor alcohols in blue cheese (34). 2-alkanols (i.e., 2-heptanol, 2-nonanol and 2-decanol), which are also important for blue cheese flavor, were only minor components in this cheese. Moreover, the proportion of alcohols in neutral volatiles (41.4%) was significantly higher in the ripened cheese than in French blue-veined varieties (<30%) (33).

3-Methyl-1-butanol, typical methyl alcohol in blue cheese deriving from the metabolism of leucine (35), was also detected in the experimental cheese. But it was not found in the control group (Supplementary Table 1). At day 33, 3-methyl-1-butanol represented 8.5% of total alcohols, a value equal to that of Gorgonzola (32), higher than Bleu des Causses (3%), but significantly lower than those measured for Bleu d'Auvergne (39%) and Roquefort (33–44%) (33).

Two aromatic alcohols (2-phenylethanol and benzyl alcohol) accumulated in the experimental cheese over time. Their concentrations increased by 118 and 32 times within 1 month, respectively (Table 4). In comparison, they were almost undetectable in the control throughout the entire maturation (Supplementary Table 1). 2-Phenylethanol is one of the most odorous alcohols in camembert and cheddar and can give cheese a very pleasant rose-like fragrance. Its appearance was previously reported to be related to the transformation of phenylalanine by yeasts (18). However, in this study, yeasts were not detected in any cheese samples. Since the control cheese was deficient in these two aromatic alcohols, the reason for their accumulation in the experimental cheese might be owed to the metabolic activity of *M*. *purpureus* GL-1. Because of their accumulation, Red-Veined Cheese showed a distinctive alcohol profile, which is characterized by a fairly high level of aromatic alcohols (77.9% of total alcohol) and a low level of 2-alkanol (3.1%). In addition, we also noticed that 2-phenylethanol and benzyl alcohol were nearly undetectable in the surface-ripened MC over the course of maturation (8). This suggests that the internal fermentation of *M*. *purpureus* GL-1 could be the main reason for the accumulation of these two aromatic alcohols.

#### Acids

A total of 15 acids were identified during the maturation period. Most of which are n-alkanoic acids. By the end of ripening, the content of acids increased 2.6 times and represented 21.8% of the total volatiles, which ranked behind ketones and alcohols. n-acids constituted 91.4% of the total acid peak area. Among them, the ones with even carbon atoms predominated and accounted for 90.8% of the total, while the ones with odd carbon numbers presented only in a tiny amount (0.7% of the total).

Three branched-chain acids (isobutyric acid, 2-methylbutanoic acid and 3-methylbutanoic acid) were detected during maturation. They are important flavor acids for blue cheese (32) and derive from the metabolism of valine, isoleucine and leucine, respectively (35). This suggests that amino acid metabolism is also one of the major pathways involved in flavor formation of Red-Veined Cheese. In comparison, no branched-chain acids were found in the control, indicating that *Monascus* fermentation may play a critical role in the formation of branched-chain acids.

#### Esters

Major flavor components of Red-Veined Cheese include esters (9.4% of total volatiles). Thirteen·esters were identified. The dominant ones were ethyl esters of free fatty acids. Compared with the control, the content of ethyl esters of free fatty acids (from C_4_ to C_10_) was significantly increased (*p* < 0.05) in the experimental cheese. Besides, we found that propyl isoamyl esters of n-acids such as isopentyl butyrate, propyl hexanoate and isopentyl hexanoate were unique to the experimental group but absent from the control (Table 4 and Supplementary Table 1). At day 33, ethyl caproate, ethyl octanoate and ethyl caprate constituted the backbone of esters profile of the experimental cheese (85.3% of total esters area), they are characteristic of mellow fruity and floral notes (32). It is reasonable to assume that these esters are also important volatiles responsible for the flavor of Red-Veined Cheese.

#### Other Compounds

Other groups of volatiles such as aldehydes, lactones and phenol represented minor fractions. No accumulation of aldehydes was observed during maturation, probably because they were reduced to the corresponding alcohols (36). Three aldehydes (nonanal, benzaldehyde and benzeneacetaldehyde) were identified in the experimental cheese at the end of ripening, among which, benzaldehyde was the most abundant, its concentration was 7 times that found in the control (Table 4 and Supplementary Table 1). Benzeneacetaldehyde with broom flower notes (32) existed only in the experimental cheese. Micro amounts of lactones and phenol were detected in the cheese headspace during ripening. In addition to providing creamy coconut notes and contribute to buttery feeling, δ-lactones can also help smooth overall cheese flavor by reducing the influence of sharpness of free fatty acids (37).

#### Sensory Analysis and Principal Component Analysis of Volatiles

As shown in Table 5, the sensory attributes of the Red-veined cheese (5, 19, and 33 days ripening) and commercial blue cheese (President Blue Cheese, from a local market) were assessed. At 19 and 33 days of storage, Red-veined cheese had a stronger mushroom note than 5 days and control 33 days. These flavor attributes had no different significantly between the 9 and 33 days of storage cheese and commercial blue cheese, but had a less odor and bitter than commercial blue cheese, which maybe related with the low level of proteolysis and lipolysis in Red-veined cheese, FAA and FFA lower than those reported for blue cheese which were mentioned above. Because The bitter taste and ammoniacal flavor and aroma, common to cheeses matured by molds, are also due to the intense proteolytic activity during the maturation process. The intense lipolytic activity also contributes greatly to the unique characteristic flavor of each type of cheese, since each resulting fatty acid has intense and different aroma and its presence speeds up the process of oxidative rancidity of lipids (38).

**Table 5:** Sensory evaluation of the Red-Veined Cheese during ripening and commercial Blue cheese (President brand).

**Attributes**	**Day 5**	**Day 19**	**Day 33**	**Control Day33^1^**	**Blue cheese**
Creamy	4.1 ± 1.2^a^	5.0 ± 0.9^a^	4.5 ± 1.0^a^	3.8 ± 1.5^a^	4.1 ± 0.8^a^
Acidity	4.5 ± 1.2^a^	4.0 ± 0.8^a^	4.9 ± 0.7^a^	4.5 ± 1.0^a^	6.2 ± 0.8^b^
Odor	3.2 ± 1.3^a^	3.8 ± 1.2^ab^	4.9 ± 1.5^ab^	3.0 ± 1.0^a^	5.4 ± 2.1^b^
Bitter	1.5 ± 0.9^a^	2.9 ± 1.2^ab^	3.1 ± 1.5^bc^	4.1 ± 0.8^bc^	5.0 ± 1.4^c^
Mushroom	1.6 ± 1.2^a^	3.7 ± 1.9^b^	2.4 ± 1.2^ab^	2.0 ± 0.9^a^	3.6 ± 1.5^b^
Salty	4.3 ± 2.1^a^	4.5 ± 0.9^a^	4.6 ± 1.2^a^	4.5 ± 0.8^a^	6.0 ± 2.1^a^

*Results are indicated as Means ± SD with different superscript lowercase letters were significantly different among ripening periods within the rows each cell (p < 0.05)*.

1 *The control without inoculation of red koji powder and ripening at 33 days*.

**Figure 4:**
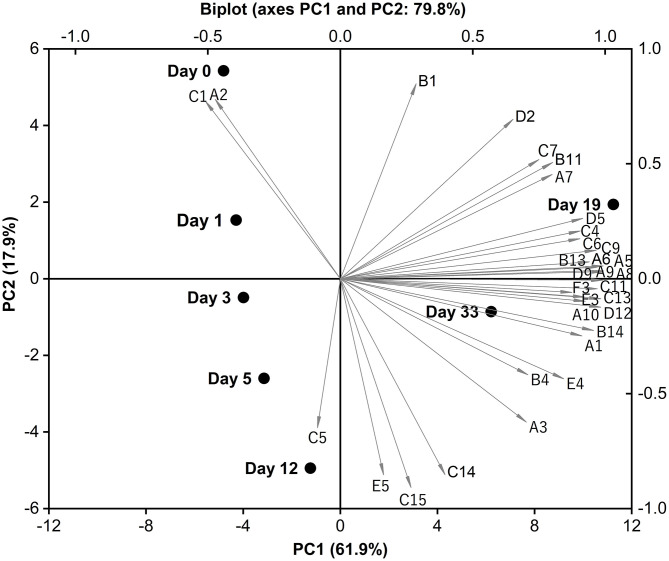
PCA analysis of the volatile profile of Red-Veined Cheese. References: A1 (Acetone), A2 (2,3-Butanedione), A3 (2-Pentanone), A5 (2-Heptanone), A6 (2-Octanone), A7 (Acetoin), A8 (2-Nonanone), A9 (8-Nonen-2-one), A10 (2-Undecanone), B1 (Ethanol), B4 (3-Methyl-1-butanol), B11 (1-Octanol), B13 (Benzyl alcohol), B14 (2-Phenylethanol), C1 (Acetic acid), C4 (Butanoic acid), C5 (2-Methylbutanoic acid), C6 (3-Methylbutanoic acid), C7 (Hexanoic acid), C9 (Octanoic acid), C11 (Decanoic acid), C13 (Dodecanoic acid), C14 (Tetradecanoic acid), C15 (Palmitic acid), D2 (Ethyl butyrate), D5 (Ethyl caproate), D9 (Ethyl octanoate), D12 (Ethyl caprate), E3 (Nonanal), E4 (Benzaldehyde), E5 (Benzeneacetaldehyde), F3 (δ-Decanolactone).

Principal component analysis (PCA) was applied as a means to visualize any relation among the volatile data of Red-Veined Cheese. All input variables in Table 3 were included in the PCA and Biplot (score plot overlapped to loadings plot) was used because it can display relationships between volatiles/cheese samples and PCs at the same time. For clarity, only compounds contributing to the main part of data variation have been identified in Figure 4. The first two principal components (PC1 and PC2) explain 79.8% of the total variance (PC1 accounts for 61.9% and PC2 for 17.9%). The experimental cheeses with different ripen time were separated on the PC1-PC2 plane. Younger samples are located on the negative PC1, while on the positive area were more ripened ones. 0-days-old cheese sample is more correlated to 2,3-Butanedione (A2) and acetic acid (C1) compared to other samples whereas a 12-days-old sample to 2-methylbutanoic acid (C5). Most of the flavor compounds that can explain data variability are concentrated at the positive extreme of PC1, mainly composed of methyl ketones, primary branched and aromatic alcohols, FFAs and straight-chain esters. This is consistent with the fact that these compounds were mostly isolated at relatively higher concentrations from cheeses ripened for 19 and 33 days. The 19-day-old sample is typically correlated to ethyl caproate (D5), whereas the main characteristic compound of 33-day-old sample was 2-phenylethanol (B14). Exploring the area between sample points of days 19 and 33, we noticed that nearly 70% of the compounds in the figure falling into this sector characterized by floral and fruity notes, mainly including methyl ketones (2-Heptanone, 2-Octanone, 2-Nonanone, 2-Undecanone), aromatic alcohols (Benzyl alcohol, 2-Phenylethanol), ethyl esters of free fatty acids (Ethyl octanoate, Ethyl caprate) and lactone (δ-Decanolactone) (39). Their amounts began to rise sharply and far exceeded the control group after about 2 weeks of ripening.

**Figure 5:**
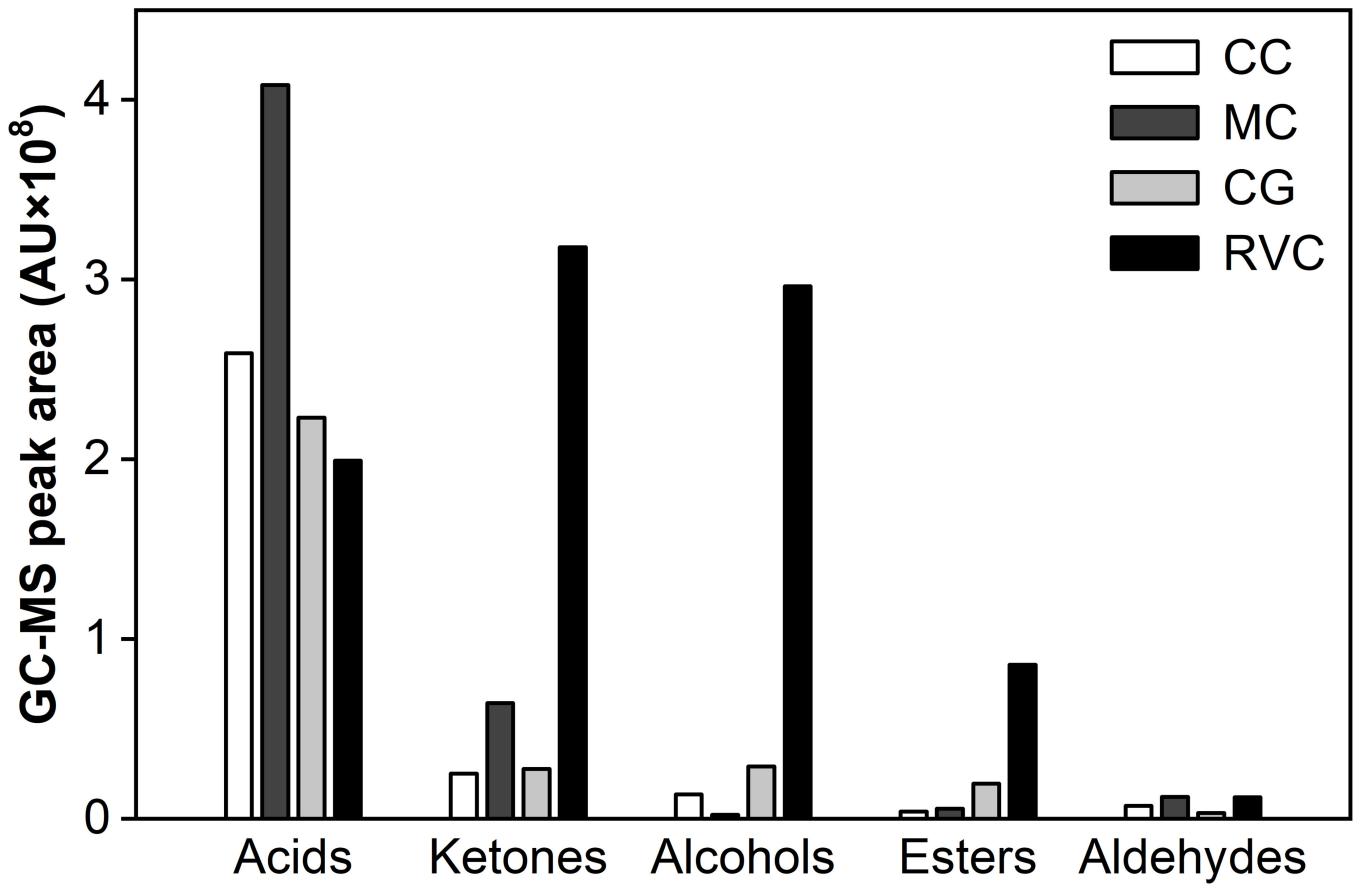
The effects of *Monascus*-fermentation on the flavor composition of Red-Veined Cheese (RVC) and surface-ripened *Monascus*-fermented cheese (MC) (8) after 33 days of ripening. CG, control group without inoculation of red koji powder; CC, control group of surface-ripened MC.

Overall, the experimental cheese was richer in volatiles. More than a quarter of compounds identified were not found in the control group (Table 4 and Supplementary Table 1). They include 3 ketones (2-pentanone, 2-hexanone,8-nonen-2-one), 3 alcohols (2-methyl-1-butanol, 3-methyl-1-butanol, benzyl alcohol), 5 acids (propanoic acid, isobutyric acid, 2-methylbutanoic acid, 3-methylbutanoic acid, nonanoic acid), 4 esters (isopentyl butyrate, propyl hexanoate, isopentyl hexanoate, ethyl nonanoate) and 1 aldehyde (benzeneacetaldehyde). Among which, benzyl alcohol and benzeneacetaldehyde impart cheese a rosy note, esters of n-acids give a sweet fruity scent, while almost all branched FFAs with sweaty smell disappeared at the end of ripening. At day 33, ten substances in the experimental group exhibited significantly higher concentrations compared with the control (*p* < 0.001), including aromatic alcohol and aldehyde (2-phenylethanol, benzaldehyde), methyl ketones (heptanone, 2-octanone, 2-nonanone, and 2-undecanone) and straight-chain esters (ethyl caproate, ethyl octanoate, ethyl caprate, and ethyl laurate). Most of them provide fruity notes. Of particular note, 2-phenylethanol, flavor alcohol with rosy aroma (39), accumulated extensively in the experimental cheese with the extension of ripening time. But it was almost undetectable in the control group. More than that, 2-phenylethanol was also undetected in surface-ripened MC (8). This indicates that the accumulation of this aromatic alcohol was associated not only with the metabolic activity of *M*. *purpureus* GL-1 but also with the growth position of the mold mycelia. During the 33 days of ripening, the total peak area of all volatiles in the experimental cheese had increased about 4-fold and finally reached 9,147.71 × 10^5^ arbitrary units, which was 3 times that of the control. At this point, ketones, alcohols, esters and aldehydes were 11.5, 10.2, 4.4, and 3.7 times that of the control, respectively. But the amount of total acids was only 89.2% of that in the control (Figure 5). Alcohols and esters, which are responsible for mild flavors, prevailed in the experimental group, together accounting for 42% of the total volatiles at the end of ripening. This value is higher than those reported for Roquefort (11–17%), Bleu des Causses (19%), Bleu d'Auvergne (24%) and Gorgonzola (39%). For acids in the ripened experimental cheese, its percentage (22%) is much below the value of blue-veined varieties (50–70%) (32, 33). In comparison, in the control group, acids predominated, accounting for 73% of the total volatiles at day 33. No significant content increases in other chemical groups were observed except for esters (Supplementary Table 1). These comparisons show clearly that it is the fermentation of *M*. *purpureus* GL-1 inside the cheese matrix that lets the ripened cheese form a distinct flavor composition, which can distinguish it from other traditional internal mold-ripened cheeses.

Additionally, when compared with the surface-ripened MC using the same strain of *M*. *purpureus* (8), more volatiles substances produced during the whole ripening time in the experimental cheese, including 6 alcohols (1-butanol, 2-methyl-1-butanol, 1-pentanol, 2-decanol, 1-octanol), 3 acids (propanoic acid, 3-methylbutanoic acid, palmitic acid), 4 esters (butyl acetate, propyl hexanoate, ethyl nonanoate, ethyl laurate) and 1 aldehyde (hexanal) (Table 4). At the end of ripening, most of them were still present in the cheese. Among which,1-pentanol, 1-octanol, 2-phenylethanol, 3-methylbutanoic acid, palmitic acid, ethyl nonanoate and ethyl laurate showed content accumulation (*p* < 0.001) from the fifth day of maturation (Table 4). Besides, two aromatic alcohols (benzyl alcohol and 2-phenylethanol) were abundant in the experimental cheese but barely detectable in surface-ripened MC (8). For flavor substances that can be detected in both types of *Monascus*-fermented cheese, straight-chain fatty acids with pungent smell (from C_2_ to C_8_) showed higher concentrations in the MC while the level of ketones with floral and fruity aroma (2-heptanone, 2-octanone, 2-nonanone, and 2-undecanone) were higher in the experimental cheese. After the same length of maturation (33 days), the amounts of alcohols, esters and ketones in the experimental cheese were 141-, 15-, and 5-fold higher, respectively, than those in surface-ripened MC. Whereas, the total acid content was only 48.8% of that found in the MC (Figure 5).

**Figure 6:**
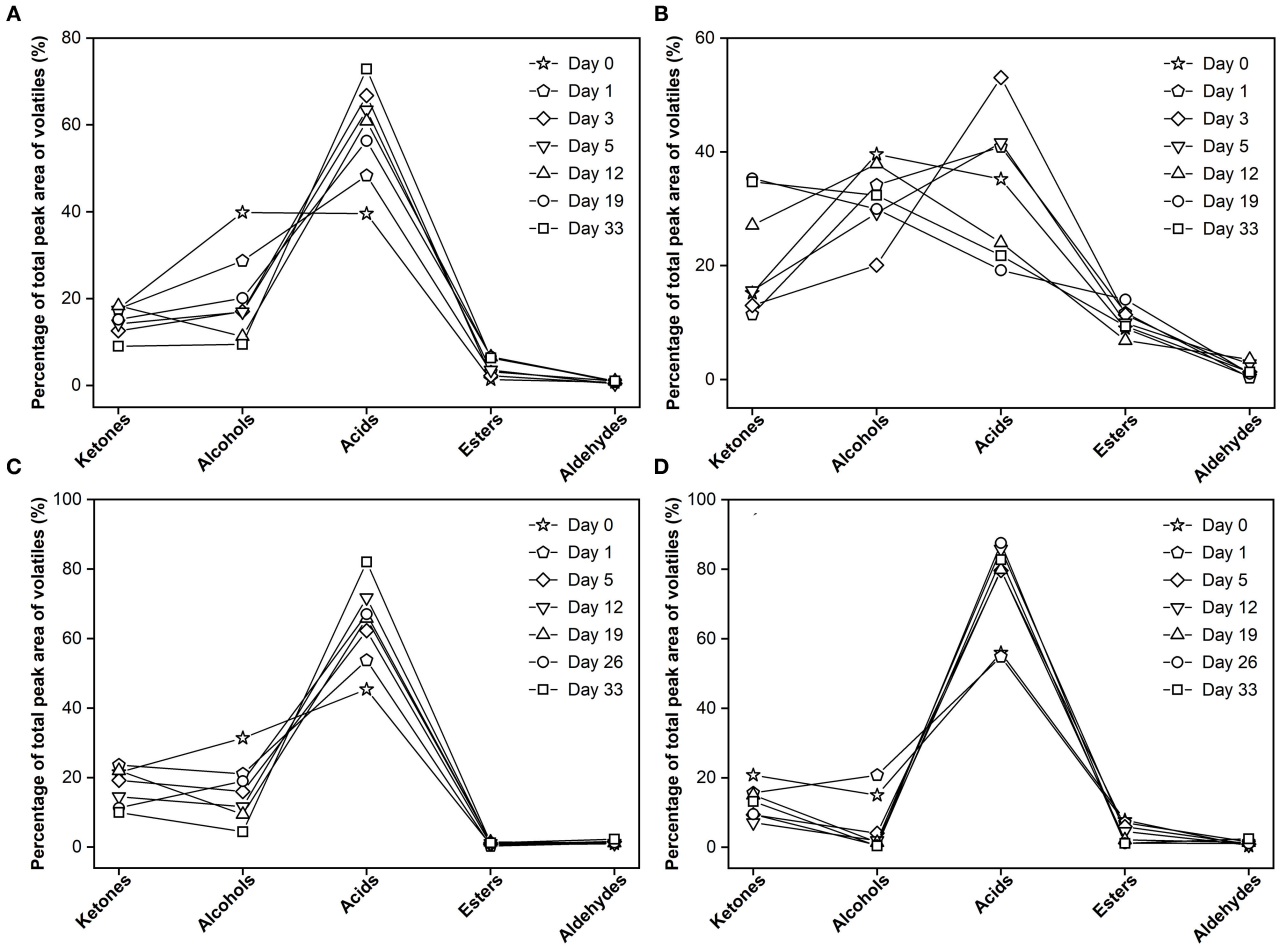
Changes in flavor profile across the 33 days. **(A)** control group without inoculation of red koji powder, **(B)** experimental group, **(C)** control group of surface-ripened *Monascus*-fermented cheese (MC) (8), **(D)** surface-ripened MC.

Another interesting finding was there were clear differences in formation between the experimental cheese and surface-ripened MC (Figure 6). At the onset of maturation, alcohols in the experimental cheese had the highest proportion among all chemical groups (39.8% of GC-MS total peak area of volatiles), acids ranked second (35.4%). As maturation continues, ketones continued to increase with its peak at day 33 (34.9%). Alcohols fluctuation over time was observed. At the end of ripening, it became the second most abundant volatile component (32.5%). Acids rose first and then dropped with its peak at day 3 (53.7%) and finally ranked third (21.9%) at day 33 (Figure 6B). In contrast, flavor development in surface-ripened MC (Figure 6D) is entirely different from that of the experimental cheese, but similar to the pattern of its control group (Figure 6C), which is characterized by a consistently high level of carboxylic acids throughout the maturation progress. The percentage of acids to the total volatiles remained the highest throughout the whole ripening period and increased from 57% at day 0 to 82.9% at day 33, while ketones, alcohols and esters decreased from 19.3, 15.3, and 8.0 to 13.1, 0.4, and 1.2%, respectively (Figure 6D). Also, the comparison revealed that the flavor formation of the control in this study (Figure 6A) was highly similar to that of the control group of the MC (Figure 6C). Since the same mold strain was employed as the red starter in this study, the difference in flavor between these two different *Monascus*-fermented varieties further confirms that the growth position of *Monascus* mycelium is a very important factor for the metabolic generation which is closely related to the formation of volatile metabolites during cheese ripening. This finding was consistent with the previous research results about *Aspergillus oryzae* (40).

## Conclusions

This is the first report on developing a novel internal mold-ripened cheese variety through the inoculation of red koji powder into the paste. It is a new *Monascus*-fermented variety that has a style similar to blue-veined cheese. Because of the bright red color of the veins, it is named as Red-Veined Cheese. Compared to the control, a higher proteolysis and lipolysis degree indicate that the use of internal *Monascus*-fermentation to accelerate cheese ripening is successful. More than that, the remarkable increase in the kind and quantity of volatile compounds also gives the experimental cheese a distinctive flavor profile, which distinguishes it not only from other internal mold-ripened cheeses but also from surface-ripened *Monascus*-fermented cheese previously reported. From a broader perspective, the result of the present study not only adds an important branch to the family tree of mold-ripened cheese but also provides a useful addition to *Monascus*-fermented food. Furthermore, this study proved that the growth position of *Monascus* mycelium might be one of the key reasons for the discrepancy in the flavor development between Red-Veined Cheese and surface-ripened *Monascus*-fermented cheese. This finding provides not only a valuable reference for flavor improvement of *Monascus*-fermented cheese but also lays a foundation for the industrialization of high-quality *Monascus*-fermented food in the future.

## Data Availability Statement

The original contributions generated for the study are included in the article/Supplementary Material, further inquiries can be directed to the corresponding author/s.

## Author Contributions

ZL and CY designed experiments. JJ and ZZ carried out experiments, analyzed experimental results and sequencing data, and wrote the manuscript. All authors contributed to the article and approved the submitted version.

## Conflict of Interest

JJ, ZZ, ZL, and CY were employed by the company Bright Dairy & Food Co., Ltd.
