# Supplementary material for: Study of the Compositional, Microbiological, Biochemical, and Volatile Profile of Red-Veined Cheese, an Internal Monascus-Ripened Variety
Source: Front Nutr. 2021 Apr 16;8:649611. doi: 10.3389/fnut.2021.649611 (PMC8085271; doi:10.3389/fnut.2021.649611)
Supplement: Supplementary file 1 [file Table_1.DOCX]

**Supplementary material**

**Study of the compositional, microbiological, biochemical and volatile profile of Red-Veined Cheese, an internal *Monascus*-ripened variety**

**Supplementary Table 1**

Volatile compounds identified in the control cheese by HS-SPME-GC-MS during the different ripening stages.

| Compounds | LRI*^b^* | IM*^c^* | Day 0 | Day 1 | Day 3 | Day 5 | Day 12 | Day 19 | Day 33 | *P^d^* |
| --- | --- | --- | --- | --- | --- | --- | --- | --- | --- | --- |
| *Ketones* |  |  |  |  |  |  |  |  |  |  |
| Acetone | 856 | MS, PI | 168.34 ± 26.95b | 171.56 ± 66.86b | 107.73 ± 15.29b | 136.77 ± 2.81b | 134.91 ± 77.28b | 515.90 ± 76.19a | 171.04 ± 34.17b | ** |
| 2-Butanone*^f^* | 893 | MS, PI | 1.02 ± 0.32 | ND*^e^* | ND | ND | ND | ND | ND |  |
| 2,3-Butanedione | 958 | MS, PI | 139.82 ± 11.56a | 46.45 ± 12.60b | 10.64 ± 6.29c | 13.02 ± 3.46c | 3.41 ± 2.65c | 21.51 ± 2.72bc | 6.03 ± 0.13c | *** |
| 2-Heptanone | 1202 | MS, PI | 5.45 ± 0.88bc | 10.02 ± 1.18bc | 2.44 ± 1.64c | 22.65 ± 11.72bc | 24.40 ± 0.45b | 51.92 ± 8.17a | 18.49 ± 0.49bc | ** |
| 2-Octanone | 1292 | MS, PI | 3.21 ± 0.40a | 0.44 ± 0.25a | 0.22 ± 0.04a | 0.61 ± 0.35a | 2.13 ± 1.44a | 5.59 ± 5.81a | 1.69 ± 0.89a | NS |
| Acetoin | 1294 | MS | 31.33 ± 14.50b | 28.97 ± 9.56b | 25.21 ± 1.43b | 18.75 ± 3.27b | 16.83 ± 0.88b | 112.72 ± 7.91a | 46.09 ± 28.33b | ** |
| 2-Nonanone | 1392 | MS, PI | 11.22 ± 2.09b | 10.89±2.28b | 4.87 ± 1.21b | 9.88 ± 4.60b | 4.83 ± 0.56b | 40.71 ± 3.02a | 27.58 ± 14.98ab | ** |
| 2-Undecanone | 1583 | MS, PI | ND | ND | 2.25 ± 0.40a | 8.15 ± 4.43a | 1.66 ± 0.25a | 7.33 ± 2.28a | 4.19 ± 1.68a | NS |
| 2-Dodecanone | 1591 | MS | ND | ND | ND | ND | 2.92 ± 1.00a | 1.93 ± 0.07a | 1.80 ± 0.04a | NS |
| 2-Decanone | 1794 | MS | ND | ND | 2.37 ± 0.75a | 1.97 ± 0.39a | 2.55 ± 1.52a | 0.67 ± 0.07a | ND | NS |
| *Alcohols* |  |  |  |  |  |  |  |  |  |  |
| Ethanol | 922 | MS, PI | 751.00 ± 126.09ab | 393.05 ± 137.52abc | 195.27 ± 69.13bc | 234.87 ± 318.71bc | 99.78 ± 1.20c | 871.43 ± 123.31a | 208.74 ± 41.40bc | ** |
| 2-Butanol*^f^* | 1023 | MS. PI | 3.37 ± 4.28 | ND | ND | ND | ND | ND | ND |  |
| 1-Butanol | 1171 | MS, PI | 1.91 ± 0.15a | 1.78 ± 1.35a | 0.23 ± 0.03a | 0.76 ± 0.21a | 0.51 ± 0.00a | 5.42 ± 5.36a | 1.69 ± 0.04a | NS |
| 1-Pentanol | 1273 | MS, PI | 2.04 ± 0.09a | 1.18 ± 0.40a | ND | ND | ND | ND | ND | NS |
| Prenol*^f^* | 1336 | MS | 4.57 ± 0.75a | 1.89 ± 0.39b | ND | ND | ND | ND | ND | * |
| 2-Heptanol | 1337 | MS | 7.02 ± 0.96ab | 3.73 ± 1.20bc | 0.52 ± 0.59c | 0.85 ± 0.01c | 0.23 ± 0.02c | 10.72 ± 0.88a | 4.06 ± 2.86bc | ** |
| 4-Methyl-2-hexanol*^f^* | 1339 | MS | ND | ND | 2.44 ± 0.09a | 1.89 ± 1.26a | 2.50 ± 0.37a | 2.63 ± 1.42a | 0.77 ± 0.11a | NS |
| 1-Hexanol | 1364 | MS, PI | 2.30 ± 0.87a | 4.66 ± 2.35a | 0.86 ± 0.25a | 1.19 ± 0.06a | 0.96 ± 0.06a | 6.34 ± 3.10a | 1.39 ± 0.74a | NS |
| 2-Ethyl-1-hexanol | 1488 | MS, PI | 2.20 ± 0.39a | ND | ND | ND | 0.57 ± 0.34a | 8.70 ± 0.72a | 8.11 ± 5.12a | NS |
| 2-Decanol | 1498 | MS, PI | 1.24 ± 0.18a | 1.29 ± 0.28a | 0.85 ± 0.34a | 0.67 ± 0.22a | 2.51 ± 0.03a | 4.75 ± 5.36a | 1.46 ± 1.81a | NS |
| 2-Nonanol | 1518 | MS, PI | 9.21 ± 1.68bc | 7.16 ± 0.68bc | 2.51 ± 0.01c | 2.90 ± 0.44bc | 2.50 ± 0.37c | 26.91 ± 1.04a | 14.55 ± 7.58b | ** |
| 1-Octanol | 1553 | MS | 27.96 ± 4.62b | 21.42 ± 3.31b | 8.47 ± 0.64b | 9.65 ± 1.14b | 8.06 ± 0.61b | 65.30 ± 0.24a | 35.02 ± 18.12b | ** |
| 1-Nonanol | 1651 | MS | ND | ND | ND | ND | 1.57 ± 0.19a | 3.06 ± 1.13a | 10.67 ± 9.03a | NS |
| 2-Phenylethanol | 1885 | MS, PI | ND | ND | ND | ND | ND | ND | 3.82 ± 0.19 |  |
| *Acids* |  |  |  |  |  |  |  |  |  |  |
| Acetic acid | 1437 | MS, PI | 467.29 ± 61.29ab | 330.67 ± 57.65ab | 236.89 ± 9.97b | 300.43 ± 84.15ab | 172.12 ± 15.71b | 844.25 ± 89.32a | 513.48 ± 369.05ab | * |
| Butanoic acid | 1608 | MS, PI | 55.80 ± 7.33b | 37.48 ± 6.85b | 17.00 ± 0.54b | 22.00 ± 3.07b | 18.06 ± 2.95b | 152.77 ± 6.50a | 72.66 ± 46.94b | ** |
| Pentanoic acid*^f^* | 1719 | MS | ND | ND | ND | ND | ND | 5.67 ± 2.82a | 1.80 ± 1.08a | NS |
| Hexanoic acid | 1823 | MS, PI | 144.58 ± 22.07b | 103.97 ± 19.59b | 53.44 ± 4.29b | 74.17 ± 13.15b | 65.31 ± 11.20b | 652.31 ± 5.83a | 407.99 ± 246.95ab | ** |
| Heptanoic acid | 1967 | MS, PI | ND | ND | ND | 1.97 ± 0.29a | 2.49 ± 0.56a | 13.78 ± 8.67a | 8.11 ± 2.57a | NS |
| Octanoic acid | 2039 | MS, PI | 89.72 ± 1.95b | 82.56 ± 12.06b | 77.25 ± 3.73b | 96.16 ± 2.32b | 89.00 ± 17.55b | 512.41 ± 13.72a | 349.14 ± 200.10ab | ** |
| Decanoic acid | 2255 | MS, PI | 49.49 ± 6.98b | 61.08 ± 9.88b | 72.40 ± 0.26b | 73.57 ± 39.58b | 70.96 ± 15.30b | 328.14 ± 7.33a | 261.82 ± 151.85ab | * |
| 9-Decenoic acid | 2315 | MS | ND | ND | ND | ND | ND | 23.16 ± 1.14a | 26.18 ± 15.18a | NS |
| Benzoic acid*^f^* | 2419 | MS | ND | ND | ND | ND | ND | 5.88 ± 6.89 | ND |  |
| Dodecanoic acid | 2466 | MS | ND | 19.12 ± 3.61c | 41.83 ± 11.50bc | 33.85 ± 4.00c | 31.32 ± 8.56c | 109.52 ± 1.28a | 102.30 ± 35.47ab | ** |
| Tetradecanoic acid | 2669 | MS | ND | 12.24 ± 2.93c | 96.20 ± 40.49bc | 236.31 ± 41.37a | 47.00 ± 9.39bc | 49.18 ± 33.93bc | 127.70 ± 10.94ab | ** |
| Palmitic acid | 2854 | MS | ND | 87.62 ± 4.62b | 231.40 ± 77.40ab | 108.46 ± 47.15b | 145.55 ± 51.17ab | 116.72 ± 26.18b | 360.42 ± 93.98a | * |
| *Esters* |  |  |  |  |  |  |  |  |  |  |
| Ethyl acetate | 886 | MS, PI | 2.10 ± 1.06c | 7.07 ± 3.75bc | 4.73 ± 0.78bc | 13.78 ± 5.05b | 38.45 ± 3.77a | 5.65 ± 0.44bc | 2.01 ± 0.39c | *** |
| Ethyl butyrate | 1032 | MS, PI | 15.06 ± 2.82bc | 14.10 ± 5.33bc | 2.04 ± 0.60c | 2.69 ± 1.05c | 2.28 ± 0.45c | 73.76 ± 6.37a | 33.05 ± 14.31b | *** |
| Butyl acetate | 1070 | MS, PI | 1.90 ± 0.74bc | 1.79 ± 0.59bc | 0.34 ± 0.01c | 0.33 ± 0.01c | 0.12 ± 0.10c | 6.76 ± 0.87a | 4.88 ± 2.15ab | ** |
| Butyl propionate*^f^* | 1151 | MS | 0.35 ± 0.03 | ND | ND | ND | ND | ND | ND |  |
| Butyl acrylate | 1194 | MS | ND | 1.46 ± 0.17a | ND | 0.24 ± 0.01a | 0.84 ± 0.07a | 9.28 ± 3.95a | 6.93 ± 3.89a | * |
| Ethyl caproate | 1253 | MS, PI | 5.56 ± 1.14c | 8.89 ± 2.59c | 3.39 ± 0.07c | 6.09 ± 1.29c | 5.44 ± 0.89c | 88.76 ± 1.96a | 44.95 ± 20.37b | *** |
| Ethyl heptanoate | 1334 | MS, PI | ND | ND | ND | ND | ND | 2.62 ± 0.17a | 4.06 ± 2.86a | NS |
| Ethyl octanoate | 1438 | MS, PI | 0.71 ± 0.21b | 7.80 ± 2.50b | 5.08 ± 0.65b | 7.29 ± 0.24b | 9.24 ± 1.33b | 67.39 ± 1.11a | 42.01 ± 20.94a | ** |
| Ethyl caprate | 1624 | MS, PI | 2.14 ± 0.75c | 6.99 ± 2.01c | 9.69 ± 0.05c | 16.78 ± 0.83bc | 9.49 ± 2.22c | 63.03 ± 3.88a | 43.66 ± 20.43ab | ** |
| Ethyl laurate | 1832 | MS | ND | ND | 2.35 ± 0.25b | 6.01 ± 2.45ab | 3.70 ± 0.66b | 15.63 ± 0.49a | 12.97 ± 5.98ab | * |
| *Aldehydes* |  |  |  |  |  |  |  |  |  |  |
| 3-Methyl butanal | 906 | MS, PI | 0.76 ± 0.13b | ND | ND | ND | ND | 9.11 ± 1.67a | 8.48 ± 0.84a | ** |
| Hexanal | 1077 | MS | 4.53 ± 0.08b | 2.18 ± 0.29c | 0.98 ± 0.11cd | 0.39 ± 0.10d | 0.92 ± 0.08cd | 7.55 ± 0.53a | 3.94 ± 0.91b | *** |
| Nonanal | 1396 | MS, PI | 8.62 ± 0.66ab | 12.59 ± 2.35ab | 3.82 ± 0.27b | 4.80 ± 1.45b | 9.28 ± 0.56ab | 15.99 ± 0.04a | 10.73 ± 5.93ab | * |
| Benzaldehyde | 1501 | MS, PI | 0.87 ± 0.34c | 1.27 ± 0.31bc | 0.85 ± 0.34c | 0.67 ± 0.22c | 0.72 ± 0.12c | 8.41 ± 0.02ab | 8.95 ± 4.85a | ** |
| *Lactones* |  |  |  |  |  |  |  |  |  |  |
| Butyrolactone | 1600 | MS | 5.69 ± 1.90ab | 1.89 ± 0.21c | 1.29 ± 0.41c | 1.42 ± 0.70c | 0.95 ± 0.16c | 7.26 ± 0.00a | 3.32 ± 0.75bc | ** |
| δ-Hexanolide*^f^* | 1766 | MS | 1.13 ± 0.18b | 0.74 ± 0.11b | 0.37 ± 0.01b | 0.63 ± 0.02b | 0.74 ± 0.17b | 3.44 ± 1.11a | 1.39 ± 0.34b | ** |
| δ-Octalactone | 1944 | MS | 2.43 ± 0.23a | 2.59 ± 0.91a | 1.55 ± 0.60a | 1.79 ± 0.04a | 4.80 ± 3.84a | 7.80 ± 0.22a | 2.69 ± 1.44a | NS |
| δ-Decanolactone | 2171 | MS | 7.32 ± 0.19a | 10.65 ± 1.74a | 7.95 ± 0.40a | 17.21 ± 3.21a | 12.00 ± 2.25a | 7.48 ± 3.83a | 26.54 ± 14.30a | NS |
| *Phenols* |  |  |  |  |  |  |  |  |  |  |
| Phenol | 1972 | MS | ND | ND | ND | 0.58 ± 0.24b | 0.33 ± 0.01b | 17.67 ± 2.84a | 2.44 ± 0.33b | ** |

*HS-SPME-GC-MS data are expressed as arbitrary units (× 10^5^) of average peak area ± standard deviations for each compound. Different superscript lowercase letters in the same row denote statistical difference (*p* < 0.05) between samples according to the Tukey test. LRI: linear retention index using a DB-WAX column; IM: identification method; *P*: probability value. NS: no significant; *: *P* < 0.05; **: *P* < 0.01; ***: *P* < 0.001; MS: mass spectra comparison using NIST library; PI: comparison with published LRI; ND: not detected.
